# Single-shot, reference-less computational wavefront sensing for complex optical fields

**DOI:** 10.1038/s41377-026-02241-5

**Published:** 2026-03-16

**Authors:** Yunhui Gao, Liangcai Cao, Din Ping Tsai

**Affiliations:** 1 Department of Electrical Engineering and State Key Laboratory of Optical Quantum Materials, City University of Hong Kong, Kowloon, Hong Kong SAR, 999077 China; 2 Department of Precision Instruments, Tsinghua University, Beijing, 100084 China; 3 State Key Laboratory of Terahertz and Millimeter Waves, City University of Hong Kong, Kowloon, Hong Kong SAR, 999077 China; 4 Department of Physics, City University of Hong Kong, Kowloon, Hong Kong SAR, 999077 China

**Keywords:** Imaging and sensing, Adaptive optics, Biophotonics, Optical metrology

## Abstract

Optical waves carry rich information in their spatial profiles and topological structures. Characterization of optical wavefronts is a key prerequisite in broad applications across fundamental research and industrial technologies. However, existing wavefront sensing techniques typically compromise between spatiotemporal resolution, compactness, and versatility. Here, we present Spatial And Fourier-domAin Regularized Inversion (SAFARI), a computational wavefront sensing approach that exploits the intrinsic physical properties such as smoothness to enable reliable reconstruction of complex wavefronts from a single exposure. Using a compact, diffuser-based wavefront sensor, we experimentally demonstrate single-shot, reference-less characterization of diverse complex wavefronts, including aberrations with up to 200 Zernike modes, structured beams carrying a topological charge of 150, and speckle fields containing more than 190,000 spatial modes. The proposed wavefront sensor offers high versatility while achieving performance comparable to or surpassing state-of-the-art task-specific solutions, making it a promising tool for coherent imaging and sensing at unprecedented resolution and complexity.

## Abstract (web summary)

We propose a high-resolution wavefront sensor that combines an optical diffuser with a computational phase retrieval algorithm, enabling the characterization of optical wavefields with unprecedented resolution and complexity.

## Graphical abstract

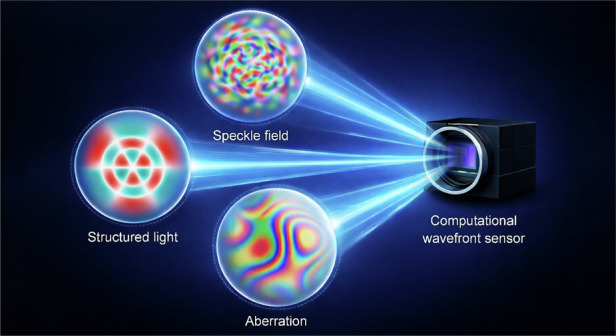

## Introduction

Quantitative characterization of optical wavefronts is a foundational technique with broad applications across optics and photonics. It serves as a key metrological tool in adaptive optics^1–3^, wavefront shaping^4–9^, beam analysis^10–12^, structured light^13–16^, industrial inspection^17–20^, and optical communication^21–23^. It also enables unconventional imaging modalities, including label-free microscopy^24–31^, fiber endoscopy^32–35^, non-line-of-sight imaging^36,37^, holographic light field imaging^38^, and imaging through scattering media^39–43^. Furthermore, it facilitates fundamental studies of light propagation and evolution in atmospheric optics^44,45^ and topological photonics^46–50^, as well as the measurement of biphoton states in quantum photonics^51–54^.

Interferometry is arguably the most classical and standard tool for coherent wave characterization^55–57^, as it enables direct access to the complex amplitude of a wavefront through interferogram analysis. However, its reliance on an additional reference beam introduces practical constraints, limiting its applicability in a wide range of experimental configurations. Alternatively, wavefront sensing can be achieved through aperture modulation^58–64^, defocus^65,66^, or translation^67–71^ diversity, or by means of shearing interferometry^72–74^. Yet, due to the ill-posed nature of phase retrieval, these methods typically require multiple measurements to ensure accurate wavefront estimation, resulting in compromised temporal resolution. Although recent strategies have sought to mitigate the effects of temporal variations^75–77^, they still depend on some degree of correlation between successive measurements, making them less ideal for characterizing rapidly evolving wavefields.

To meet the requirements of most wavefront sensing applications, single-shot, reference-less measurement schemes have been developed. For example, Shack-Hartmann wavefront sensors (SHWFSs) use a microlens array to measure the local phase gradient of the wavefront based on focal spot displacements^49,78,79^. More recently, a broader class of wavefront sensors, which we refer to here as generalized SHWFSs, has adopted a similar principle but replaces the microlens array with alternative encoding optics, such as designed phase masks^80^, aperture arrays^81^, diffusers^82–89^, or cross gratings^90–94^, offering enhanced spatial resolution and dynamic range. Fourier-based approaches^95^, exemplified by pyramid wavefront sensors^96^ and partitioned aperture wavefront sensors^97^, have demonstrated strong potential for achieving high-resolution and high-sensitivity wavefront sensing in both astronomical^98^ and microscopic applications^99–101^. Based on the principle of diversity measurement, it is also possible to encode multi-shot information into a single capture through multiplexing or parallel acquisition^102–106^. Additionally, wavefront sensing has been achieved in various scenarios using coherent diffractive imaging^107–111^, speckle-correlation scattering matrix^112–115^, angular-sensitive nanophotonic devices^116–118^, optical neural networks^119,120^, or sensorless approaches^121–125^. Despite significant advancements, most existing solutions are primarily designed for mildly aberrated wavefronts and face challenges under extreme conditions involving strong aberrations, scintillations, or dense singularities^126–128^. Deep learning-based algorithms have been explored to boost performance across different sensor configurations^129–134^, albeit at the cost of generalizability and versatility.

**Fig. 1:**
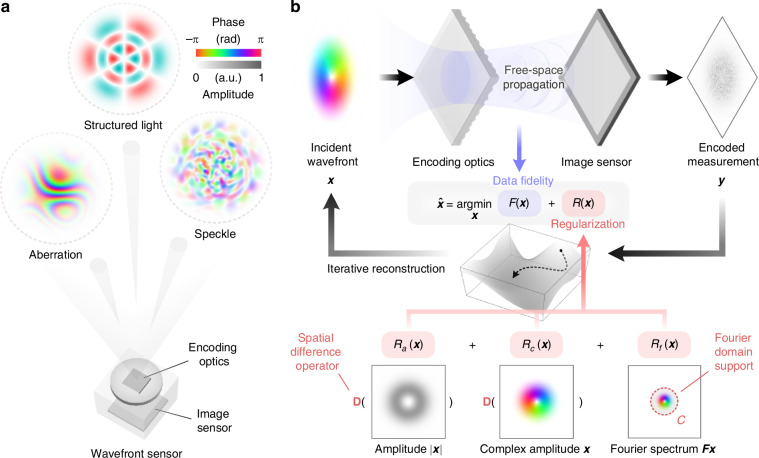
Overview of the proposed wavefront sensing framework. **a** The wavefront sensor is capable of characterizing diverse types of optical wavefronts, such as aberrations, structured beams, and speckle fields, etc., while maintaining a compact form factor. **b** Wavefront reconstruction workflow of SAFARI, which involves solving an inverse problem constrained by both the forward model and multiple physical priors in the spatial and Fourier domains

Here, we present a single-shot, reference-less computational wavefront sensing approach for characterizing ultra-complex optical fields. By leveraging the intrinsic physical properties of optical wavefronts, we introduce a general computational framework, termed Spatial And Fourier-domAin Regularized Inversion (SAFARI), to reliably reconstruct wavefronts from a single exposure. When combined with a diffuser-based wavefront sensor, SAFARI enables accurate characterization of a wide range of complex optical fields, including highly aberrated or turbulent wavefronts, structured light with ultra-high topological charges, and speckle fields containing dense singularities. The complexity of these wavefronts, quantified by the number of contributing Zernike modes, topological charge, or speckle density, exceeds the capability of state-of-the-art wavefront sensors. As illustrated in Fig. 1a, the SAFARI-empowered wavefront sensor exhibits a compact form factor, high versatility, and high spatial resolution, while achieving performance that is comparable to, or even superior to, that of task-specific solutions.

## Results

### Computational wavefront sensing via SAFARI

In a typical wavefront sensor, the relationship between the incident wavefront profile ***x*** and the measured field amplitude ***y*** can be described by a generalized forward model of coherent optical imaging systems:

1 $${\boldsymbol{y}}=| {\boldsymbol{A}}{\boldsymbol{x}}|$$

where ***A*** denotes the measurement matrix of the system. Recovering the complex wavefront from a single exposure is an ill-posed problem known as phase retrieval^135–137^, and thus requires exploiting additional prior knowledge about the wavefront. To address this, we propose SAFARI as a simple yet effective computational framework for complex wavefront reconstruction. As illustrated in Fig. 1b, SAFARI employs joint spatial and Fourier-domain regularization, leading to the following inverse problem:

2 $$\hat{{\boldsymbol{x}}}=\mathop{{\rm{a}}{\rm{r}}{\rm{g}}{\rm{m}}{\rm{i}}{\rm{n}}}\limits_{{\boldsymbol{x}}}\,\{\mathop{\underbrace{{\Vert |{\boldsymbol{A}}{\boldsymbol{x}}|-{\boldsymbol{y}}\Vert }_{2}^{2}}}\limits_{F({\boldsymbol{x}})}+\mathop{\underbrace{{\lambda }_{a}{\Vert {\boldsymbol{D}}|{\boldsymbol{x}}|\Vert }_{2}^{2}}}\limits_{{R}_{a}({\boldsymbol{x}})}+\mathop{\underbrace{{\lambda }_{c}{\Vert {\boldsymbol{D}}{\boldsymbol{x}}\Vert }_{2}^{2}}}\limits_{{R}_{c}({\boldsymbol{x}})}+\mathop{\underbrace{{I}_{C}({\boldsymbol{x}})}}\limits_{{R}_{f}({\boldsymbol{x}})}\}$$

where *F*(***x***) is the data-fidelity loss term ensuring consistency with the forward model in Eq. (1). *R*_*a*_(***x***) and *R*_*c*_(***x***) represent the spatial-domain regularization terms for the amplitude and complex amplitude, respectively, where ***D*** is the spatial finite-difference operator, and *λ*_*a*_ and *λ*_*c*_ are the corresponding regularization weights. These regularization terms promote smooth wavefront profiles in terms of both amplitude and complex amplitude. *R*_*f*_(***x***) is the Fourier-domain regularization term, with *I*_*C*_ denoting the {0, + *∞*}-valued indicator function of set *C*, which is defined as

3 $$C\mathop{=}\limits^{{\rm{def}}}\left\{{\boldsymbol{x}}={{\boldsymbol{F}}}^{-1}{\boldsymbol{u}}\,| \,u({k}_{x},{k}_{y})=0,{k}_{x}^{2}+{k}_{y}^{2} > {k}_{\max }^{2}\right\}$$

where ***F*** denotes the Fourier transform, *k*_*x*_ and *k*_*y*_ represent the coordinates in the spatial frequency domain, and ${k}_{\max }$ defines the radius of the support region. Essentially, *R*_*f*_ enforces a strict circular support boundary in the Fourier domain and leads to a band-limited reconstruction. In all experiments, we empirically set the Fourier support radius ${k}_{\max }$ to be 1/4 of the image sensor’s Nyquist frequency, leading to an oversampling ratio of 64/*π* ≈ 20. This choice represents a balance between sensor bandwidth utilization and the need to ensure the well-posedness of the phase retrieval problem. The introduction of these regularization terms is motivated by the empirical observation that most wavefronts exhibit smooth spatial distributions with energy predominantly concentrated in low-frequency components. The spatial and Fourier-domain regularizers can be interpreted as *soft* and *hard* low-pass filters and, when combined into a unified framework, offer flexibility in handling different types of wavefronts with varying degrees of distortion (see Supplementary Note 1). The resulting nonconvex and nonsmooth optimization problem in Eq. (2) can be solved via standard optimizers such as the proximal gradient algorithm, as detailed in “Materials and methods”. A MATLAB implementation of the algorithm is available at ref. ^138^.

A noteworthy feature of SAFARI is its direct recovery of the wavefront complex amplitude, which differs from the phase gradient reconstruction commonly used in generalized SHWFSs^139,140^. It naturally handles phase wraps, discontinuities, and singularities without requiring specialized processing. While post-processing methods have been proposed to address phase singularities in gradient-based measurements^141–145^, they often impose constraints on vortex density and wavefront complexity to ensure accurate reconstruction.

### Experimental demonstration with a diffuser-based wavefront sensor

**Fig. 2:**
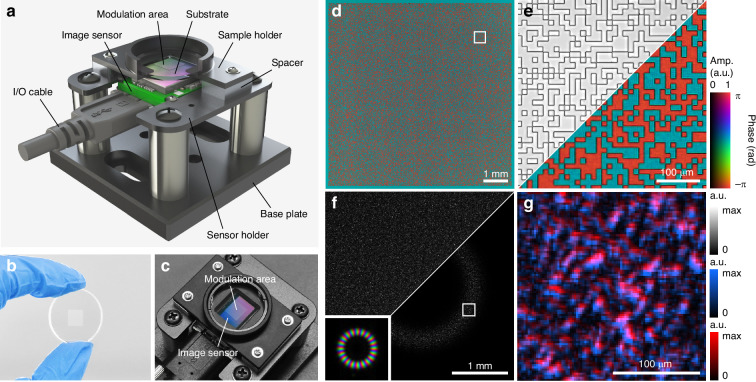
Design and calibration of the wavefront sensor. **a** Schematic illustration of the sensor configuration. **b** and **c** correspond to the photographs of the fabricated DOE sample (**b**) and the prototype wavefront sensor (**c**), respectively. **d** DOE transmission function calibrated in situ via ptychography. **e** Enlarged region of interest in (**d**), where a brightfield microscope image (upper left) is also shown for comparison. **f** Captured raw intensity images under plane wave (upper left) and LG_20,0_ beam (lower right) illumination. The LG_20,0_ beam profile is shown in the inset. **g** Comparison between the two intensity profiles in the enlarged region of interest in (**f**), where the blue and red color maps are used to indicate the intensity profiles under plane wave and LG_20,0_ beam illumination, respectively

We demonstrate SAFARI with a diffuser-based wavefront sensor, as shown in Fig. 2. Diffusers have been widely studied in the context of computational imaging^146–150^. In wavefront sensing and coherent imaging, in particular, they have been popular due to their high manufacturability, broad spectral compatibility^151–153^, and superior wavefront encoding efficiency^154–159^, which can be partly explained by the introduction of randomness in compressive sensing and machine learning theory^160,161^. In the prototype wavefront sensor shown in Fig. 2a–c, a diffractive optical element (DOE) is mounted a few millimeters in front of an image sensor. For this specific optical configuration, the forward model of Eq. (1) can be reformulated as follows (Supplementary Note 2):

4 $${\boldsymbol{y}}=| {\boldsymbol{Q}}{\rm{diag}}({\boldsymbol{m}}){\boldsymbol{x}}|$$

where ***A*** = ***Q***diag(***m***), with ***m*** and ***Q*** denoting the transmission function of the DOE and the free-space propagation operator from the DOE to the image sensor, respectively. More specifically, the measurement operator ***A*** consists of a DOE modulation step, which is calculated via an element-wise product with the DOE transmission function, and a free-space propagation step, which is calculated based on the angular spectrum model^162^. The DOE is designed to function as a thin diffuser, introducing random binary phase modulation with a feature size of 12 μm, a total dimension of 7.2 mm × 7.2 mm, and a phase retardance of *π* at the design wavelength of 532 nm. The complex transmission profile of the DOE is calibrated in situ using ptychography, as described in “Materials and methods” and shown in Fig. 2d, e. The random DOE profile scrambles the incident wavefront, and the raw image captured by the sensor resembles a speckle pattern. Although appearing random, the speckle pattern encodes both amplitude and phase information of the wavefront, which can be interpreted from the perspective of the optical memory effect^163^. Figure 2f, g illustrates this encoding mechanism by comparing raw intensity patterns from a plane wave and an LG_20,0_ beam (Laguerre–Gaussian (LG) beam with azimuthal order 20 and radial order 0) illumination. The overall intensity envelope of the image reflects the intensity distribution of the incident wavefront, while the transverse displacement of the speckle pattern reveals the local phase gradient. Under certain approximations, this relationship has been exploited in previous works to directly retrieve the wavefront^86,87^. In contrast, our approach employs a more accurate scalar diffraction theory for diffraction modeling, as given by Eq. (4). The image sensor in our prototype system has a pixel size of 2.4 μm, and the central 2800 × 2800 pixels are selected as the imaging field of view (FOV), which is slightly smaller than the DOE modulation region. Given the 1/4 bandwidth utilization of the support constraint, the theoretical half-pitch spatial resolution of the wavefront sensor is 9.6 μm, which is four times the pixel size. The resulting number of independent phase sampling points is 700 × 700. Accordingly, the maximum numerical aperture of the incident wavefront is limited to 0.028 to ensure accurate and reliable reconstruction. As demonstrated in Supplementary Note 3, while SAFARI can retrieve wavefronts whose spectral profiles slightly exceed the Fourier support, spectral filtering prior to measurement may be necessary when the incident wavefront carries a significant amount of high-frequency components beyond the wavefront sensor’s bandwidth.

**Fig. 3:**
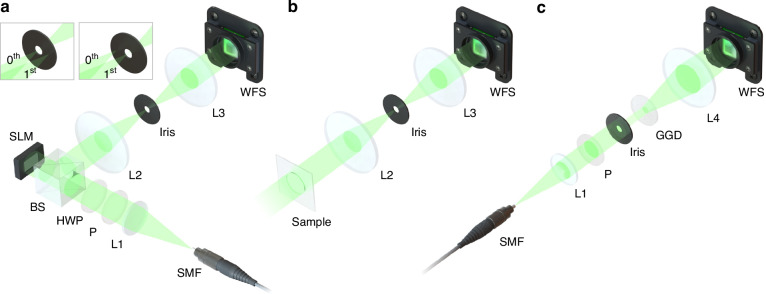
Experimental setups. **a** Experimental setup for characterizing phase-only or complex amplitude wavefronts. The zeroth (left inset) and first (right inset) orders of the diffracted wave are selected by an iris at the Fourier plane to perform phase-only modulation (e.g., for synthetic aberrations and turbulence) and complex amplitude modulation (e.g., for structured beams), respectively. **b** Experimental setup for characterizing transmissive amplitude samples. **c** Experimental setup for characterizing speckle fields. SMF single-mode fiber, L lens, P polarizer, HWP half-wave plate, BS beam splitter, SLM spatial light modulator, WFS wavefront sensor, GGD ground glass diffuser

To evaluate the performance and demonstrate the versatility of SAFARI, we experimentally characterized the key parameters of the wavefront sensor and conducted wavefront sensing experiments on three representative classes of wavefronts, including synthetic aberrations and turbulence, structured light with phase discontinuities or singularities, and complex speckle fields with densely distributed vortices. The experimental setups are shown in Fig. 3 and Supplementary Fig. 1, with detailed descriptions provided in the following sections. The wavefront reconstruction accuracy was quantitatively evaluated by comparing the results with ground-truth wavefront profiles measured using ptychography (see “Materials and methods”).

### Performance characterization of the wavefront sensor

**Fig. 4:**
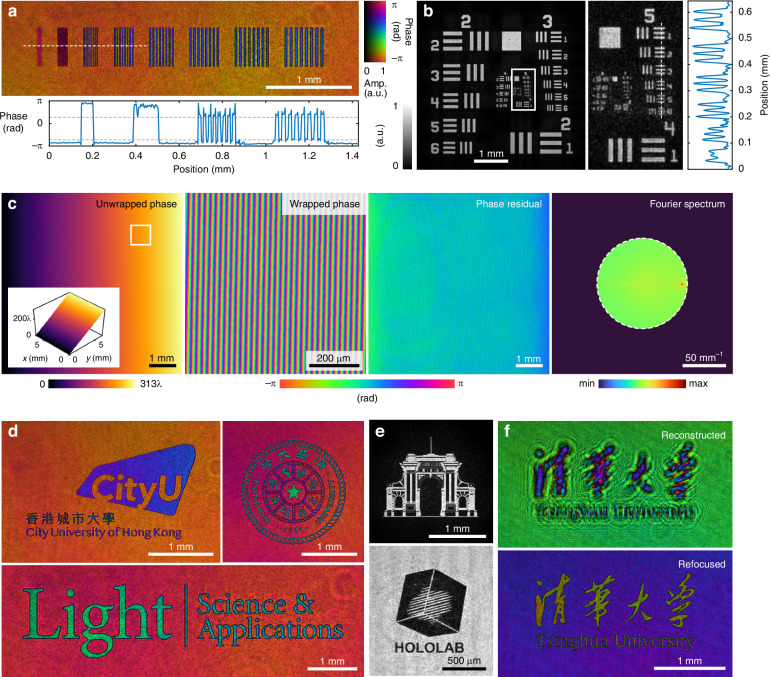
Performance characterization of the wavefront sensor. **a** Reconstructed complex amplitude of a phase resolution pattern, where the line widths vary from 1 to 8 SLM pixels from left to right, and the finest resolvable line width is 11.22 μm, corresponding to 3 SLM pixels. The cross-sectional phase profile is shown below, where the range between the two dashed lines indicates the theoretical phase retardance of *π*. Note that the reconstructed phase values of the first two groups of line patterns deviate from the theoretical values due to aliasing effects. **b** Reconstructed amplitude and the amplitude cross-sectional profile of a negative 1951 USAF amplitude resolution test target, where the finest resolvable line width is 8.77 μm (Group 5, Element 6). **c** Wavefront reconstruction of a tilted plane wave. From left to right: unwrapped phase profile within a 2800 × 2800 FOV, enlarged FOV of the wrapped phase profile corresponding to the boxed region, phase residual map compared to the reference ptychographic measurement, and Fourier modulus (in logarithmic scale) of the reconstructed wavefront, where the dashed circle indicates the support region. **d** Reconstructed complex amplitude of representative phase objects. **e** Reconstructed amplitude profiles of negative (upper) and positive (lower) amplitude objects. **f** Demonstration of autofocusing, where the directly reconstructed (upper) and refocused (lower) wavefronts are shown (Supplementary Video 1)

We first performed imaging of phase and amplitude objects to characterize the performance of the wavefront sensor. The experimental setups to measure phase and amplitude objects are shown in Fig. 3a, b, respectively. For the phase objects, a phase-only reflective spatial light modulator (SLM) was used to generate designed phase patterns, which were relayed to the wavefront sensor through a 4f system. The setup for imaging amplitude objects is identical, except that the reflective SLM is replaced with a transmissive sample coated with low-reflectivity chrome patterns. Figure 4a shows the reconstruction of a phase resolution chart, which is a binary phase pattern with different line widths generated by the SLM. The spatial resolution of the wavefront sensor is between 2 and 3 SLM pixels, corresponding to a range between 7.48 and 11.22 μm. Figure 4b shows the reconstructed amplitude of a negative 1951 USAF resolution test target, where Group 5 Element 6 is resolved, corresponding to a line width of 8.77 μm. Both experiments show agreement with the theoretical resolution of the wavefront sensor.

Another critical design specification for wavefront sensors is the dynamic range, defined as the maximum peak-to-peak variation of the incident wavefront. To test the dynamic range of our wavefront sensor, we generated tilted plane waves by displaying blazed gratings on the SLM. Figure 4c shows the reconstructed wavefront of a tilted plane wave with a dynamic range of approximately 313 wavelengths, equivalent to 166 μm in optical path length, approaching the theoretical bandwidth limit of the Fourier support. The phase residual was calculated by comparison with the ptychographic measurement, yielding a root mean square (RMS) error of 0.260 rad, corresponding to an optical path length of 0.0414 wavelengths.

To further demonstrate the high-resolution imaging capability, we performed wavefront imaging of various phase and amplitude objects, as shown in Fig. 4d, e, respectively. Both positive and negative amplitude objects were successfully reconstructed, although imaging the latter is often considered challenging for phase microscopy and wavefront sensing. It is also worth noting that the high spatial resolution and holographic imaging capability of the wavefront sensor enable autofocus of the reconstructed wavefront, eliminating the need for precise image focus adjustment during acquisition, as illustrated in Fig. 4f. The autofocusing process is visualized in Supplementary Video 1.

### Wavefront sensing for aberrations and turbulence

**Fig. 5:**
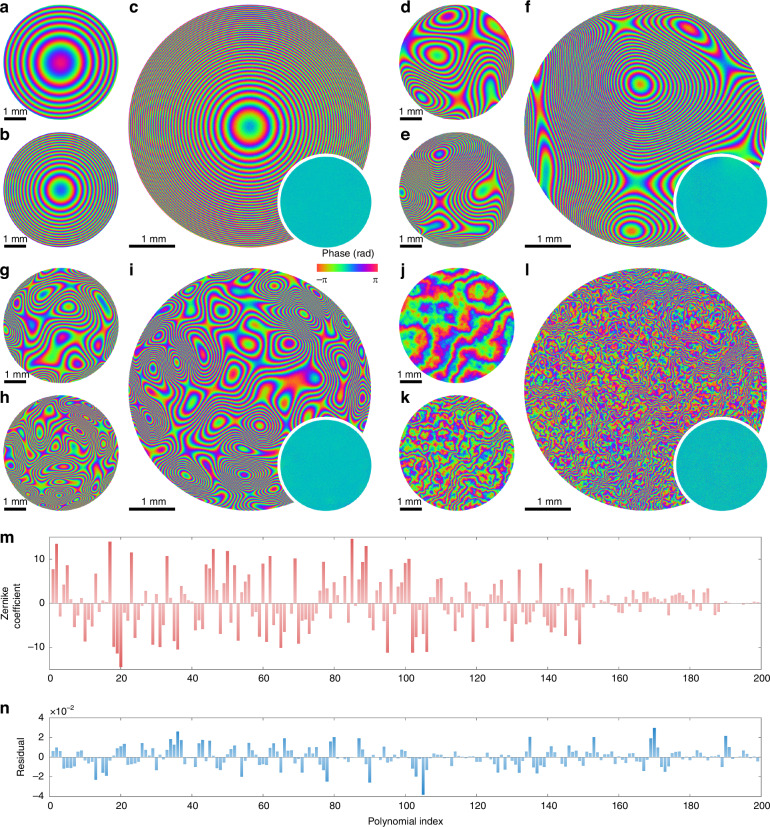
Wavefront sensing application for synthetic aberrations and turbulence. **a**–**c** Reconstruction of parabolic phase profiles with a focal length of 1000 mm (**a**), 500 mm (**b**), and 200 mm (**c**). **d**–**f** Reconstruction of synthetic aberrations using Zernike polynomials with increased dynamic range. **g**–**i** Reconstruction of synthetic aberrations using Zernike polynomials with increased mode numbers. **j**–**l** Reconstruction of synthetic turbulence generated via Fourier phase screens with increased complexity. The insets in (**c**, **f**, **i**, **l**) show the phase residuals with respect to the reference measurement. **m**, **n** Zernike mode decomposition of the phase profile in (**i**), where the mode coefficients (**m**) and residuals (**n**) for the first 200 orders are shown following the OSA/ANSI convention. Note that the first term ${Z}_{0}^{0}$ is omitted owing to the global phase ambiguity

We performed wavefront sensing of synthetic aberrations and turbulence, which are commonly encountered in optical metrology and adaptive optics applications. As shown in Fig. 3a, the SLM is used to generate controlled wavefront distortion, which is then measured by the wavefront sensor located at the conjugate plane of a 4f system. Figure 5a–c shows the reconstructed parabolic phase profiles with varying focal lengths of 1000, 500, and 200 mm, respectively. Given the reconstructed field $\widehat{{\boldsymbol{x}}}$ and the ground truth ***x***_ref_ measured by ptychography, we quantify reconstruction accuracy using phase residual maps (inset in Fig. 5c), calculated as $\arg (\widehat{{\boldsymbol{x}}}\odot {\bar{{\boldsymbol{x}}}}_{{\rm{ref}}})$, where ⊙ denotes element-wise multiplication, and ${\bar{{\boldsymbol{x}}}}_{{\rm{ref}}}$ denotes the complex conjugate of ***x***_ref_. We then tested the imaging of more complex synthetic aberrations, whose phase profiles were generated using the following equation:

5 $$\phi =2\pi s\cdot \mathop{\sum }\limits_{i}{c}_{i}{\phi }_{i}$$

where *ϕ*_*i*_ denotes the *i*th Zernike polynomial following the OSA/ANSI index^164^, *c*_*i*_ denotes the corresponding coefficient sampled uniformly from [−1, 1], and *s* is a scaling factor that controls the dynamic range of the wavefront. Figure 5d–f, g–i shows three representative reconstructed wavefronts with increasing dynamic range (21 Zernike modes with *s* = 4, 8, and 12) and spatial resolution (66, 136, and 231 Zernike modes with *s* = 2), respectively. We further performed Zernike mode decomposition on the unwrapped phase in Fig. 5i, and the resulting coefficients and residuals are shown in Fig. 5m, n, respectively. Note that the coefficients of higher-order modes are smaller because the reconstructed wavefronts were slightly cropped to avoid boundary artifacts. In addition to Zernike aberrations, we also performed wavefront sensing of synthetic atmospheric turbulence generated using the Fourier-based phase screens^165,166^. Owing to the experimental limitations, only single-layer phase screens with varying Fried’s coherence length *r*_0_ = 0.3, 0.1, and 0.03 mm were addressed to the SLM, as shown in Fig. 5j–l, respectively. Simulation studies on reconstructing highly scintillated atmospheric turbulence can be found in Supplementary Note 3. The twelve test cases achieved an average RMS error of 0.0330 wavelengths (Supplementary Table 1). Supplementary Fig. 2 shows the unwrapped phases of the aberrations and the corresponding Fourier magnitudes (i.e., the point spread functions). A maximum dynamic range of 280 rad was demonstrated. The reconstruction also accounts for inevitable SLM imperfections, such as phase discontinuities resulting from the wrapped phase modulation and a certain degree of amplitude modulation (Supplementary Fig. 3).

### Wavefront sensing for structured light

Structured light has emerged as a powerful tool across multiple disciplines owing to its rich spatial features and unique topological properties^167^. These wavefronts typically exhibit intricate intensity variations as well as phase singularities and discontinuities that pose challenges to conventional wavefront sensing techniques. Recently, deep learning methods have been proposed to realize singularity detection and mode decomposition for certain classes of structured light^168–170^. Here, we show that our general-purpose wavefront sensor achieves competitive performance while maintaining broad applicability across diverse structured light fields.

**Fig. 6:**
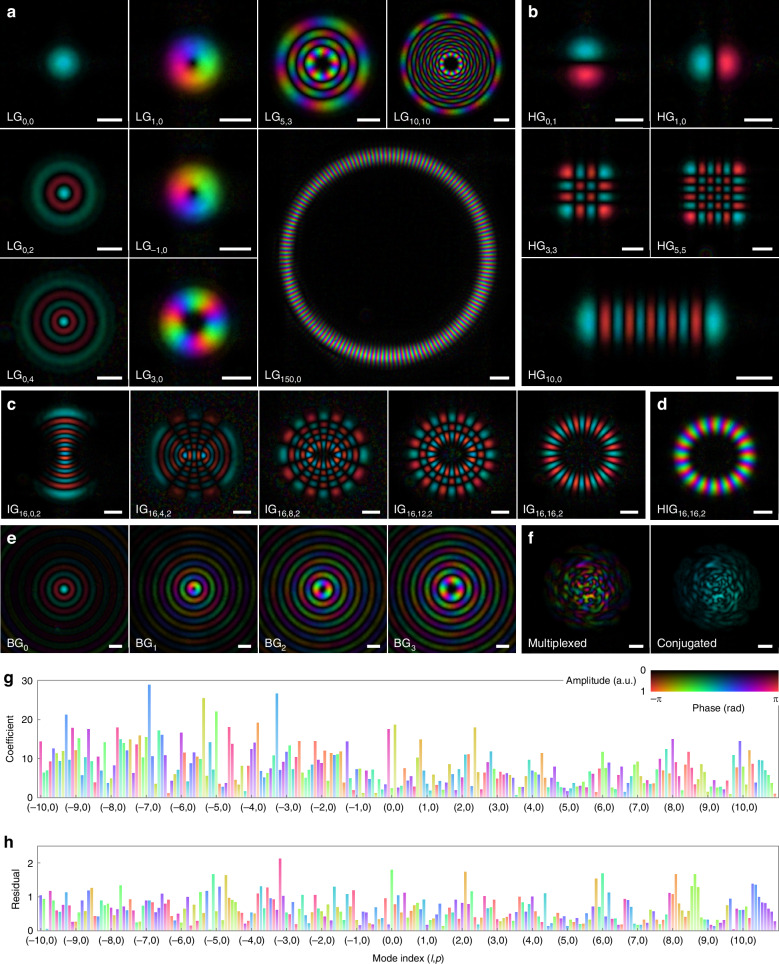
Wavefront sensing application for structured light. **a** Representative Laguerre–Gaussian beams LG_*l*,*p*_, where *l* and *p* denote the azimuthal and radial orders, respectively. **b** Representative Hermite-Gaussian beams HG_*m*,*n*_, where *m* and *n* are the mode indices. **c** Representative Ince-Gaussian beams IG_*p*,*m*,*ϵ*_, where *p*, *m*, and *ϵ* denote the order, degree, and ellipticity parameters, respectively. **d** A helical Ince-Gaussian beam, which is the linear combination of even and odd IG beams ${{\rm{HIG}}}_{p,m,\epsilon }={{\rm{IG}}}_{p,m,\epsilon }^{e}+{\rm{j}}\cdot {\,{\rm{IG}}}_{p,m,\epsilon }^{o}$. **e** Bessel-Gaussian beams BG_*l*_, where *l* denotes the azimuthal order. **f** A multiplexed Laguerre–Gaussian beam (left) and its phase-conjugated profile with respect to the reference measurement (right). **g**, **h** LG mode decomposition of (**f**) for −10≤*l*≤10 and 0 ≤ *p* ≤ 10, where the magnitude and color-coded phase of the mode coefficients (**g**) and residuals (**h**) are shown. Note that for the HG and IG beams, the tip, tilt, and piston phase terms have been compensated via least-square fitting using the uniform-phase region for visualization purposes. Scale bars, 500 μm

We employ the same overall experimental setup shown in Fig. 3a to generate structured light. Compared with aberration generation, the only modification is that the phase patterns addressed to the SLM are designed to achieve complex amplitude modulation, and the first diffraction order, rather than the zeroth, is selected by the iris to obtain the desired beam profiles^171^. Figure 6a presents the reconstructed complex amplitudes for various LG modes. Complete characterization of the transverse field profile enables straightforward identification of topological charges and further analysis of beam quality and mode purity. It is worth noting that we have demonstrated reconstruction of LG beams with topological charges up to *l* = 150. In fact, the upper limit here is constrained primarily by the optical system’s ability to generate higher-order LG beams with sufficient purity, rather than by the performance of the wavefront sensor. We also characterized other common classes of structured beams, including Hermite-Gaussian beams (Fig. 6b), Ince-Gaussian beams (Fig. 6c), helical Ince-Gaussian beams (Fig. 6d), and Bessel-Gaussian beams (Fig. 6e). Figure 6f shows the reconstructed wavefront of a multiplexed beam containing 231 LG modes (−10 ≤ *l* ≤ 10, 0 ≤ *p* ≤ 10). The uniform phase of the phase-conjugated wavefront $\widehat{{\boldsymbol{x}}}\odot \exp (-{\rm{j}}\arg {{\boldsymbol{x}}}_{{\rm{ref}}})$ confirms accurate complex field reconstruction. Additionally, we quantify the intensity similarity between the retrieved and ground-truth fields using the intensity correlation coefficient *κ*^57,110,170,172^:

6 $$\kappa =\frac{{\sum }_{i}({a}_{i}-{\mu }_{a})({b}_{i}-{\mu }_{b})}{\sqrt{{\sum }_{i}{({a}_{i}-{\mu }_{a})}^{2}{\sum }_{i}{({b}_{i}-{\mu }_{b})}^{2}}}$$

where *a*_*i*_ and *b*_*i*_ denote the intensity values of the estimated and reference wavefronts for pixel *i*, respectively, with *μ*_*a*_ and *μ*_*b*_ representing their mean values. The correlation coefficient for the multiplexed wavefront is 0.9927. Figure 6g, h shows the calculated magnitude and phase of the mode coefficients and their residuals, respectively.

### Wavefront sensing for speckle fields

**Fig. 7:**
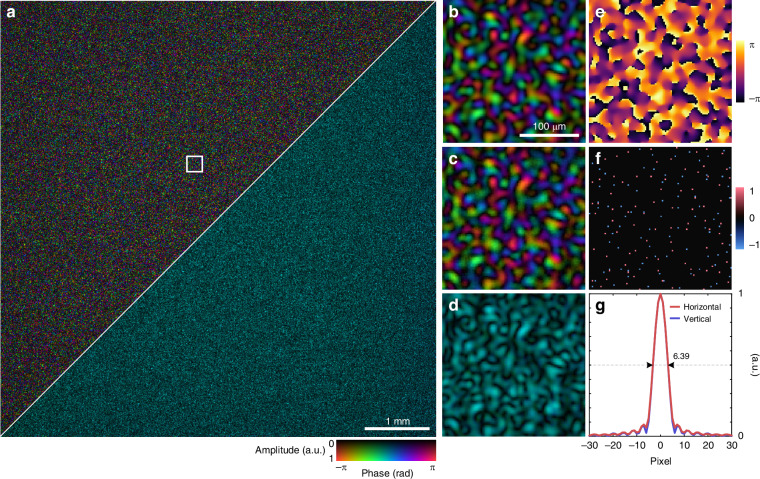
Wavefront sensing application for complex speckle fields. **a** Full-field-of-view holographic reconstruction of a speckle field containing approximately 192,000 spatial modes (upper left) and the corresponding phase-conjugated wavefront (lower right). **b**–**d** Enlarged region of interest in (**a**). Results obtained via SAFARI (**b**), ptychographic measurements (**c**), and the phase-conjugated wavefront (**d**) are shown. **e** Phase profile of (**b**). **f** Calculated topological charge map reveals the phase singularities in (**e**). **g** Horizontal and vertical cross sections of the autocorrelation of the speckle field profile in (**a**). The average FWHM is 6.39 pixels, which approximately corresponds to the average speckle grain size

As a final demonstration, we applied our wavefront sensor to characterize complex speckle fields, which exhibit random amplitude and phase distributions with densely distributed phase singularities. Such fields typically arise when light propagates through disordered media, such as biological tissue or multimode fibers, or is reflected from diffuse surfaces. The experimental setup to obtain and measure speckle fields is shown in Fig. 3c. A ground glass diffuser is illuminated by a collimated laser beam, and a speckle field is obtained at the Fourier plane and measured by the wavefront sensor. Figure 7 shows the reconstructed wavefront of a dense speckle field. Again, the phase-conjugated wavefront $\widehat{{\boldsymbol{x}}}\odot \exp (-{\rm{j}}\arg {{\boldsymbol{x}}}_{{\rm{ref}}})$ maintains a uniform phase across the entire FOV. To quantify the complexity of the speckle field, we calculate the number of spatial modes within the FOV by dividing the total number of pixels by the average speckle grain size, which is estimated according to the full width at half maximum (FWHM) of the autocorrelation function of the field profile^8^. As shown in Fig. 7g, the measured FWHM is 6.39 pixels, corresponding to approximately 192,000 spatial modes in a 2800 × 2800 FOV. The number and density of resolvable spatial modes exceed those of state-of-the-art approaches by more than an order of magnitude (Supplementary Note 4). We also tested imaging of speckle fields with varying numbers of modes by adjusting the aperture size of the iris placed in front of the diffuser (Supplementary Fig. 4).

To further validate the reconstruction accuracy, we performed an additional experiment with an amplitude resolution target placed immediately downstream of the diffuser, as illustrated in Supplementary Fig. 5a. We then applied the Fourier transform to the reconstructed speckle field, and the resulting Fourier spectrum, shown in Supplementary Fig. 5f, g, accurately reproduces the target’s structural features. These results confirm the high accuracy and spatial resolution of the wavefront sensor, which could potentially be used to enable holographic imaging of diffuse objects.

## Discussion

In summary, we have introduced SAFARI as a versatile computational framework for wavefront reconstruction. It captures the intrinsic physical priors of complex optical fields by leveraging simple yet effective spatial and Fourier-domain regularization techniques. By combining SAFARI with a compact, diffuser-based wavefront sensor, we experimentally realized single-shot, reference-less characterization of diverse types of optical wavefronts. Specifically, we demonstrated the reconstruction of highly aberrated wavefronts composed of approximately 200 Zernike modes, structured light carrying a topological charge up to *l* = 150, and speckle fields containing more than 190,000 spatial modes. The most prominent feature of our wavefront sensing approach is its unprecedented generalizability while maintaining comparable or even superior performance to existing task-specific, state-of-the-art solutions.

At the technical level, the proposed wavefront sensing scheme can be interpreted as a combination of coherent diffractive imaging techniques and a generalized SHWFS, enabling direct, high-resolution holographic reconstruction in a compact form factor. Supplementary Note 4 presents a quantitative comparison with existing wavefront sensing techniques. The proposed wavefront sensor achieves the largest number of resolvable modes and the highest spatial resolution, while maintaining performance comparable to commercial SHWFSs and quadriwave lateral shearing interferometry wavefront sensors in terms of dynamic range and accuracy. Based on our experimental results, we anticipate that the proposed wavefront sensor will be readily applicable to a wide range of applications, including beam characterization, profilometry, and phase microscopy. Its compact design, high spatial resolution, and ability to characterize complex wavefields make it a particularly promising tool for the study of structured light^167,173^, label-free microscopy^29^, wavefront shaping^174^, and speckle metrology^175^, where existing techniques often face trade-offs between system complexity and information throughput.

Similar to many computational imaging approaches, the proposed wavefront sensing scheme is more computationally intensive than conventional wavefront sensors and may not yet meet real-time application requirements. Additionally, due to the inherent ill-posedness and nonconvexity of the optimization problem, parameter tuning is needed to achieve optimal performance in challenging scenarios (Supplementary Note 3). Nevertheless, it is expected that advanced physics-based deep learning frameworks integrating physical models with data priors can accelerate reconstruction and improve robustness^176,177^. Another possible direction for further improvement is the image sensor’s bandwidth utilization. In our current implementation, an oversampling ratio of approximately 20 is adopted, which is considerably larger than the theoretical limit for phase retrieval. Improvements to the diffuser design, modeling, and calibration methods could potentially relax the experimental requirement, enabling higher spatial resolution that is competitive with other quantitative phase imaging modalities.

Although SAFARI has been demonstrated using a diffuser-based wavefront sensor, we emphasize its broader compatibility with generalized SHWFSs (Supplementary Note 3). Furthermore, by leveraging advancements in encoding optics^178–183^ and detection schemes^184,185^, we anticipate that SAFARI could be potentially extended to characterize higher-dimensional optical fields with multiple coherent states^186–195^.

## Materials and methods

### Experimental setup

As shown in Fig. 2a, the wavefront sensor consists of a bare-board complementary metal-oxide-semiconductor (CMOS) image sensor (Alvium 1800 U-2050m, Allied Vision) and a DOE sample placed approximately 3.6 mm above it. The image sensor has a pixel pitch of 2.4 μm and a full resolution of 5496 × 3672 pixels. The DOE is designed to provide random binary phase modulation with a feature size of 12 μm, a total dimension of 7.2 mm × 7.2 mm, and a phase retardance of *π* at 532 nm. The sample is fabricated on a glass substrate with a 1-inch diameter. The CMOS image sensor and the DOE sample are then integrated by custom optomechanical components made by computer numerical control machining. In all experiments, the wavefront sensor is mounted on a two-axis motorized translation stage (MTS25/M-Z8, Thorlabs) that can move in the transverse plane for system calibration and for acquiring the ground-truth wavefront via ptychography.

The experimental setup for synthetic phase object, aberration, turbulence, and structured light characterization is shown in Fig. 3a. The light source is a 532 nm fiber-coupled solid-state laser (MGL-III-532-200mW, Changchun New Industries Optoelectronics Technology). The coherent light from a single-mode fiber is collimated by a plano-convex lens with a focal length of 100 mm and adjusted to the desired linear polarization state through a polarizer and a half-wave plate. A reflective, phase-only liquid-crystal-on-silicon SLM (GAEA-2, HOLOEYE) with a pixel size of 3.74 μm and a resolution of 4094 × 2464 pixels is used to generate the target wavefronts. The SLM has been calibrated in advance to ensure a linear phase response between 0 and 2*π* (Supplementary Note 5). The SLM plane is relayed to the detection plane of the wavefront sensor using a 4f system consisting of two plano-convex lenses with a focal length of 200 mm. A variable iris is placed at the Fourier plane of the 4f system to select the desired diffraction order. Specifically, the zeroth order is selected for synthetic phase objects, aberrations, and turbulence, whereas the first order is selected for structured light.

The experimental setup for amplitude object imaging is shown in Fig. 3b, which is kept identical to that used for phase object imaging (Fig. 3a), except that the reflective phase-only SLM is replaced with transmissive amplitude samples. The amplitude samples used in the experiment are either a standard negative 1951 USAF resolution test target (R1DS1N, Thorlabs) or self-fabricated samples consisting of positive or negative patterned chrome coatings on glass substrates.

The experimental setup for speckle field characterization is shown in Fig. 3c. The diameter of the linearly polarized laser beam is controlled by a variable iris. A 220-grit ground-glass diffuser (DG10-220-MD, Thorlabs) is placed downstream to generate speckle patterns. The diffuser and the wavefront sensor are placed approximately at the front and back focal planes of a plano-convex lens with a focal length of 200 mm, respectively.

### Reconstruction algorithm

Wavefront reconstruction involves minimizing the objective function in Eq. (2), which is essentially a nonsmooth and nonconvex optimization problem. The problem can be readily solved by the proximal gradient algorithm, which proceeds by alternating between gradient updates with respect to the differentiable terms *F*(***x***) + *R*_*a*_(***x***) + *R*_*c*_(***x***) and proximal updates with respect to the nondifferentiable term *R*_*f*_(***x***)^196^. Here, we adopt an accelerated variant of the proximal gradient algorithm^197^:

7 $${{\boldsymbol{v}}}^{(i)}={{\boldsymbol{u}}}^{(i-1)}-\gamma {\nabla }_{{\boldsymbol{u}}}\left(F({{\boldsymbol{u}}}^{(i-1)})+{R}_{a}({{\boldsymbol{u}}}^{(i-1)})+{R}_{c}({{\boldsymbol{u}}}^{(i-1)})\right)$$

8 $${{\boldsymbol{x}}}^{(i)}={{\rm{prox}}}_{\gamma {R}_{f}}({{\boldsymbol{v}}}^{(i)})={{\mathcal{P}}}_{C}({{\boldsymbol{v}}}^{(i)})$$

9 $${{\boldsymbol{u}}}^{(i)}={{\boldsymbol{x}}}^{(i)}+{\beta }_{i}({{\boldsymbol{x}}}^{(i)}-{{\boldsymbol{x}}}^{(i-1)})$$

where *i* = 1, 2,… is the iteration number, *γ* is the step size, ***u***^(0)^ = ***x***^(0)^ is the initial estimate, and ${\beta }_{i}=\frac{i}{i+3}$ is the extrapolation parameter. The proximal operator with respect to the indicator function of set *C*, by definition, corresponds to the projection operator ${\mathcal{P}}$ onto *C*. A detailed analytical derivation and convergence properties of the algorithm can be found in Supplementary Notes 6 and 7, respectively. The algorithm typically converges within a few hundred iterations. For wavefronts with a dimension of 1000 × 1000, this takes 5–10 s on a laptop computer with an Intel Core i9-12900HK @2.90 GHz CPU and an NVIDIA GeForce RTX 3080 Ti GPU, as shown in Supplementary Fig. 6.

### Design and fabrication of the DOE sample

The DOE in the wavefront sensor serves as a key encoding element for the incident wavefronts and is designed to function as a thin diffuser. A binary pattern is adopted to minimize fabrication difficulty. A phase retardance of *π* is employed to maximize intensity contrast in the raw measurements, a choice that can be partly attributed to the improved conditioning for phase-gradient encoding^198^. The feature size of the DOE pattern is selected as a trade-off between encoding efficiency and sampling requirements. Specifically, a smaller feature size produces higher intensity contrast and greater randomness, whereas larger features are easier to calibrate and introduce fewer modeling errors. In this work, we empirically chose a feature size of 12 μm, corresponding to approximately five times the sensor pixel size, which is sufficiently large for accurate sensor sampling while still providing adequate intensity contrast.

The DOE sample was fabricated using photolithography via a commercial service provided by Chengdu Zhilan Micro-Nano Technology Co., Ltd. During fabrication, a photoresist layer was first spin-coated and solidified onto the quartz substrate. Subsequently, a photomask prepared according to the designed pattern was positioned on top of the photoresist layer, and a contact-mode exposure was performed. After exposure, the photoresist was developed, during which the exposed regions of the photoresist were dissolved and removed, while the unexposed regions remained intact, thereby forming the desired pattern in the photoresist. The pattern was transferred onto the substrate via reactive ion etching. Finally, the residual photoresist was stripped off, completing the entire fabrication process.

### Calibration of the DOE transmission function

Unlike most speckle tracking-based reconstruction methods, our approach adopts a more accurate forward model based on scalar diffraction theory, which requires precise knowledge of the DOE transmission function. Previously, the DOE profile was either predefined or measured using interferometric methods, which could introduce practical issues such as fabrication or alignment errors and experimental complexity. To overcome these limitations, we developed a reference-less in situ calibration method inspired by recent advances in coded ptychography^199^. As illustrated in Supplementary Fig. 7a, during calibration, an additional diffuser is placed in the light path to introduce random illumination. The wavefront sensor is then translated laterally across a two-dimensional grid of 9 × 9 positions in the transverse plane, and a series of intensity images are captured. The forward model can be expressed as

10 $${{\boldsymbol{y}}}_{k}=| {\boldsymbol{Q}}{\rm{diag}}({\boldsymbol{m}}){{\boldsymbol{T}}}_{k}{\boldsymbol{p}}|$$

where ***p*** denotes the illumination wavefront, ***T***_*k*_ and ***y***_*k*_ denote the lateral translation operator and the captured amplitude image for the *k*th measurement, respectively. The relative positions can be determined with subpixel accuracy through image registration using the reference region outside the DOE modulation area, as illustrated in Supplementary Fig. 7c, d. The translational diversity from a total of *K* = 81 measurements introduces sufficient data redundancy for the joint recovery of both the probe and the DOE profile, achieved by solving the following multivariate optimization problem:

11 $$\widehat{{\boldsymbol{m}}},\widehat{{\boldsymbol{p}}}=\mathop{{\rm{argmin}}}\limits_{{\boldsymbol{m}},{\boldsymbol{p}}}\frac{1}{K}\mathop{\sum }\limits_{k=1}^{K}{\left\Vert | {\boldsymbol{Q}}{\rm{diag}}({\boldsymbol{m}}){{\boldsymbol{T}}}_{k}{\boldsymbol{p}}| -{{\boldsymbol{y}}}_{k}\right\Vert }_{2}^{2}$$

A modified stochastic gradient descent algorithm is employed to solve Eq. (11), as derived in detail in Supplementary Note 7. The in situ calibration scheme enables high-resolution and robust characterization of the DOE profile, thereby obviating the experimental difficulties associated with precise optical alignment.

### Reference wavefront characterization via ptychography

For all wavefront sensing experiments presented in this work, we measure the reference wavefront as the ground truth using ptychography^69^. Similar to the calibration experiment, the wavefront sensor is translated laterally, and a sequence of intensity images is captured, as illustrated in Supplementary Fig. 7b. The inverse problem is formulated as

12 $${{\boldsymbol{x}}}_{{\rm{ref}}}=\mathop{{\rm{argmin}}}\limits_{{\boldsymbol{x}}}\frac{1}{K}\mathop{\sum }\limits_{k=1}^{K}{\left\Vert | {\boldsymbol{Q}}{\rm{diag}}({\boldsymbol{m}}){{\boldsymbol{T}}}_{k}{\boldsymbol{x}}| -{{\boldsymbol{y}}}_{k}\right\Vert }_{2}^{2}$$

and is solved by a stochastic gradient descent algorithm (Supplementary Note 7). Unlike during calibration, the diffuser transmission function ***m*** is known in this case, and a smaller *K* (e.g., 5 × 5 scanning positions) is usually sufficient to yield stable recovery. The ptychographic phase retrieval algorithm is initialized with the results from SAFARI, allowing fast convergence within a few iterations. Since the same wavefront sensor is used for both single-shot and ptychographic measurements, the field reconstructed by SAFARI $\widehat{{\boldsymbol{x}}}$ and the reference field ***x***_ref_ share the same sampling conditions and spatial resolution, enabling straightforward image registration and quantitative comparison.

## Supplementary information

Supplementary Video 1

Supplementary Information

## Data Availability

The data that support the findings of this study are available from the corresponding authors upon reasonable request.

## Code availability

The code for wavefront reconstruction is available at ref. ^138^.
