# Supplementary material for: Single-shot, reference-less computational wavefront sensing for complex optical fields
Source: Light Sci Appl. 2026 Mar 16;15:174. doi: 10.1038/s41377-026-02241-5 (PMC12993084; doi:10.1038/s41377-026-02241-5)
Supplement: Supplementary file 2 — Supplementary Information [file 41377_2026_2241_MOESM2_ESM.pdf]

Supplementary information for

Single-shot, reference-less computational wavefront sensing for complex optical fields

Yunhui Gao[1,2], Liangcai Cao[2*], Din Ping Tsai[1,3,4*]

[1]Department of Electrical Engineering and State Key Laboratory of Optical Quantum Materials, City University of Hong Kong, Kowloon, Hong Kong SAR, 999077, China.
[2]Department of Precision Instruments, Tsinghua University, Beijing, 100084, China.
[3]State Key Laboratory of Terahertz and Millimeter Waves, City University of Hong Kong, Kowloon, Hong Kong SAR, 999077, China.
[4]Department of Physics, City University of Hong Kong, Kowloon, Hong Kong SAR, 999077, China.

*Corresponding author(s). E-mail(s): clc@tsinghua.edu.cn (L. Cao); dptsai@cityu.edu.hk (D. P. Tsai);

Table of Contents

Supplementary Figures

Supplementary Fig. 1 Photographs of the experimental systems.
Supplementary Fig. 2 Unwrapped phase and the Fourier magnitude of the reconstructed wavefronts.
Supplementary Fig. 3 Reconstructed amplitude and phase of synthetic aberrations.
Supplementary Fig. 4 Experimental characterization of speckle fields with varying densities.
Supplementary Fig. 5 Validation of the accuracy of speckle field reconstruction.
Supplementary Fig. 6 Runtime evaluation of SAFARI based on simulation.
Supplementary Fig. 7 Ptychographic measurement for the DOE transmission function and ground-truth wavefronts.

Supplementary Tables

Supplementary Table 1 Phase reconstruction errors for aberrations and turbulence.

Supplementary Notes

Supplementary Note 1 Mathematical interpretation of SAFARI.
Supplementary Note 2 Modeling of the wavefront sensor.
Supplementary Note 3 Quantitative evaluation of SAFARI.
Supplementary Note 4 Comparison with existing wavefront sensing methods.
Supplementary Note 5 Calibration of the spatial light modulator.
Supplementary Note 6 Algorithm derivation and convergence theory.
Supplementary Note 7 Ptychographic reconstruction algorithms.

Supplementary Videos

Supplementary Video 1 Visualization of the autofocusing process.

Supplementary Figures

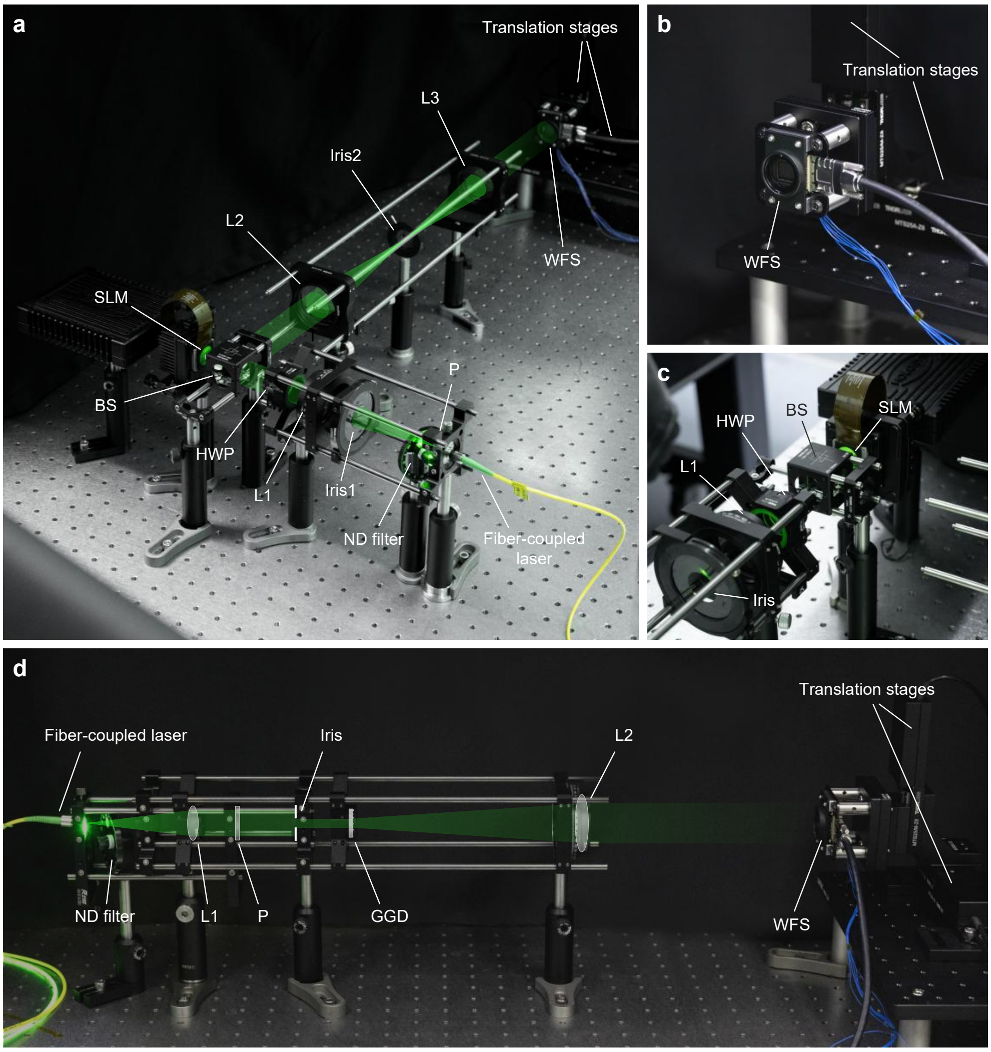

Supplementary Fig. 1 Photographs of the experimental systems. (**a**) Experimental setup for characterizing phase objects, aberrations and structured beams. (**b**, **c**) Enlarged views of (**a**). (**d**) Experimental setup for characterizing speckle fields. ND filter, neutral-density filter.

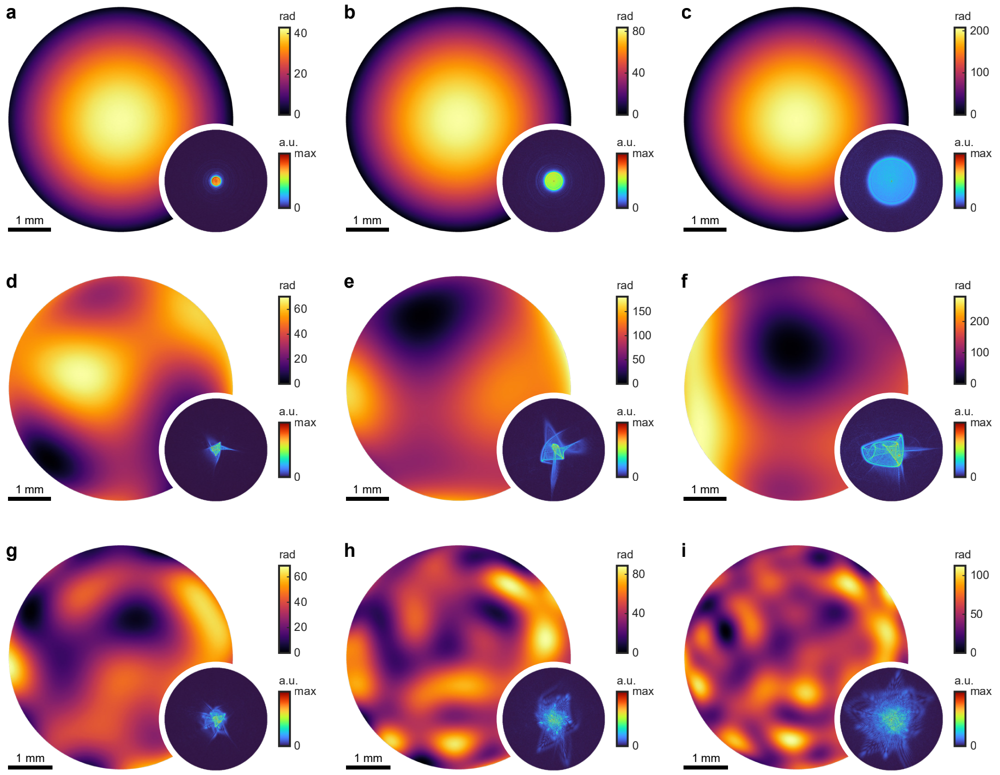

Supplementary Fig. 2 Unwrapped phase and the Fourier magnitude of the reconstructed wavefronts. (**a-c**), (**d-f**), and (**g-i**) correspond to the wavefronts in Fig. 5a-c, d-f, and g-i, respectively. Only Fourier spectra within the support region are shown in the insets.

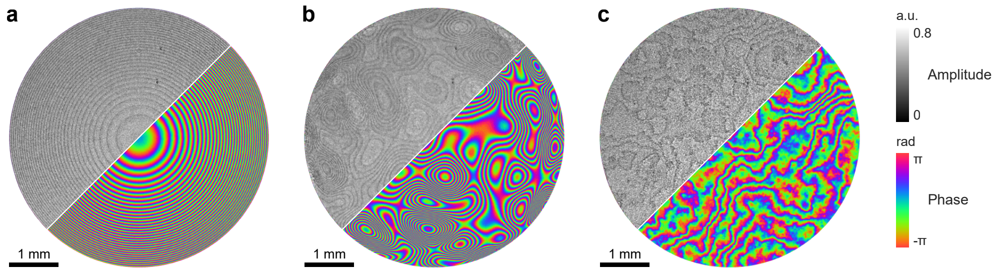

Supplementary Fig. 3 Reconstructed amplitude and phase of synthetic aberrations. (**a**), (**b**) and (**c**) are three representative wavefronts corresponding to Fig. 5c, i, and k, respectively.

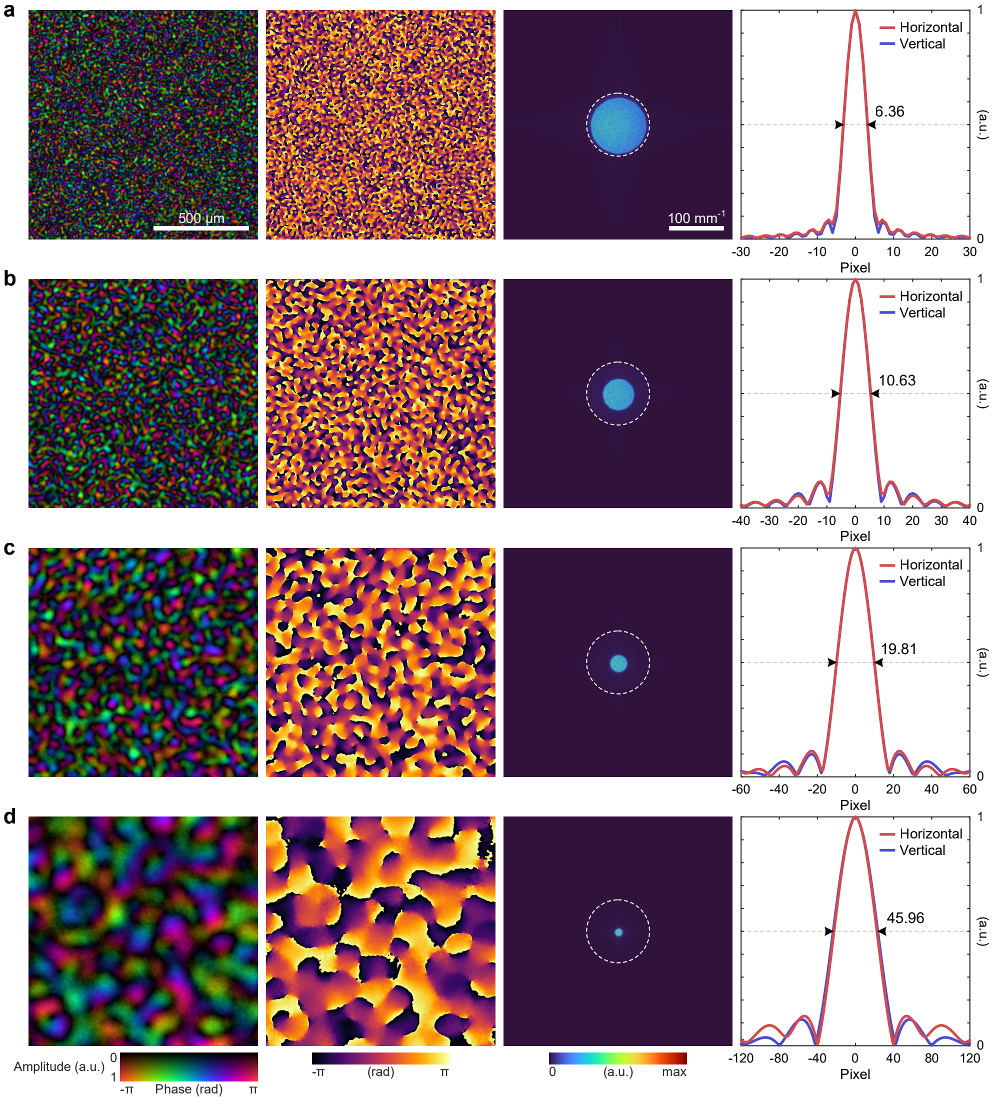

Supplementary Fig. 4 Experimental characterization of speckle fields with varying densities. From left to right: reconstructed complex amplitude, phase, Fourier magnitude, and the cross section of the autocorrelation of the field profile. The dashed circle indicates the support region in the Fourier domain. (**a-d**) Four representative speckle fields with different speckle densities are shown, where the number of modes within the entire 2,800×2,800 FOV is 194,123 (**a**), 69,382 (**b**), 19,988 (**c**), and 3,713 (**d**), respectively. Note that only 500 × 500 enlarged regions of interest are shown for better visibility.

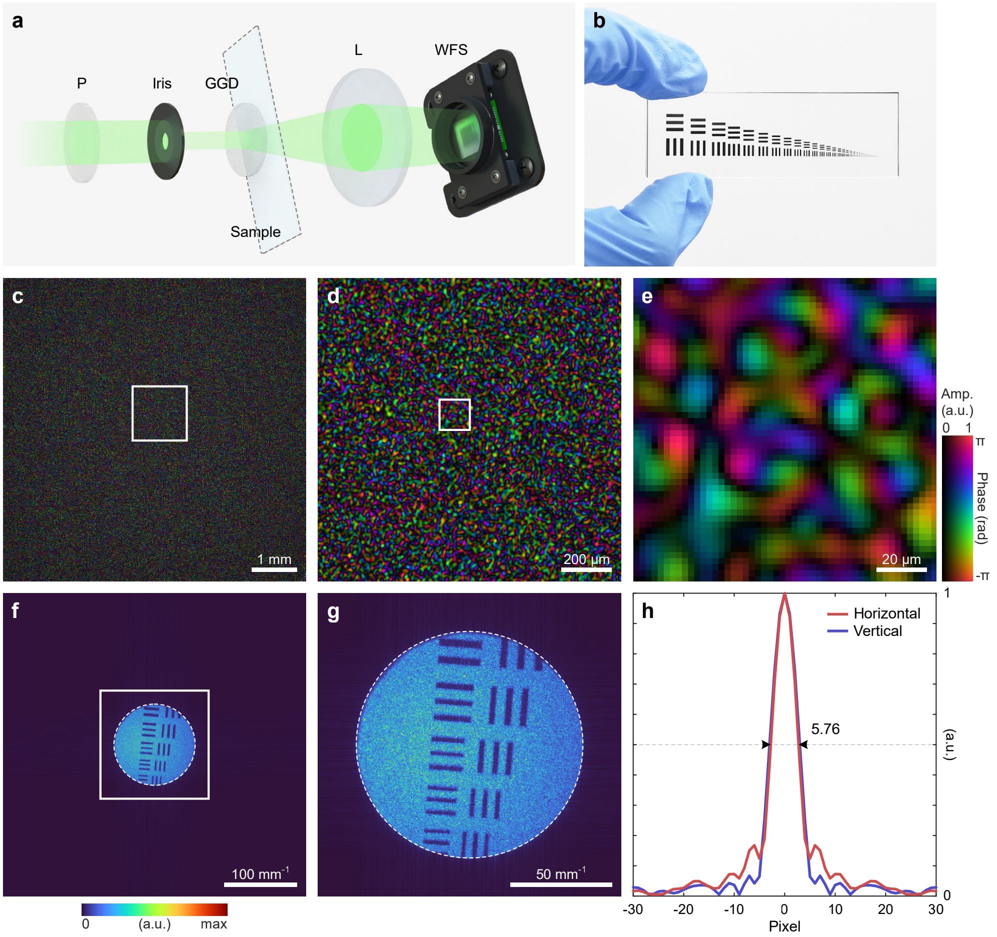

Supplementary Fig. 5 Validation of the accuracy of speckle field reconstruction. (**a**) Experimental setup, which is the same as Fig. 3c except that an additional sample is placed after the ground glass diffuser. (**b**) Photograph of the amplitude resolution target used as the imaging sample. (**c-e**) Holographic reconstruction of the speckle field. (**c**) 2,800×2,800 full-FOV field profile. (**d**) 500×500 enlarged FOV of the boxed region in (**c**). (**e**) 50×50 enlarged FOV of the boxed region in (**d**). (**f**,**g**) Fourier magnitude of the wavefront, where (**g**) shows the enlarged view of the boxed region in (**f**). The dashed circles indicate the support region. (**h**) Cross sections of the autocorrelation of the speckle field. The number of spatial modes is estimated to be 236,768 in this case.

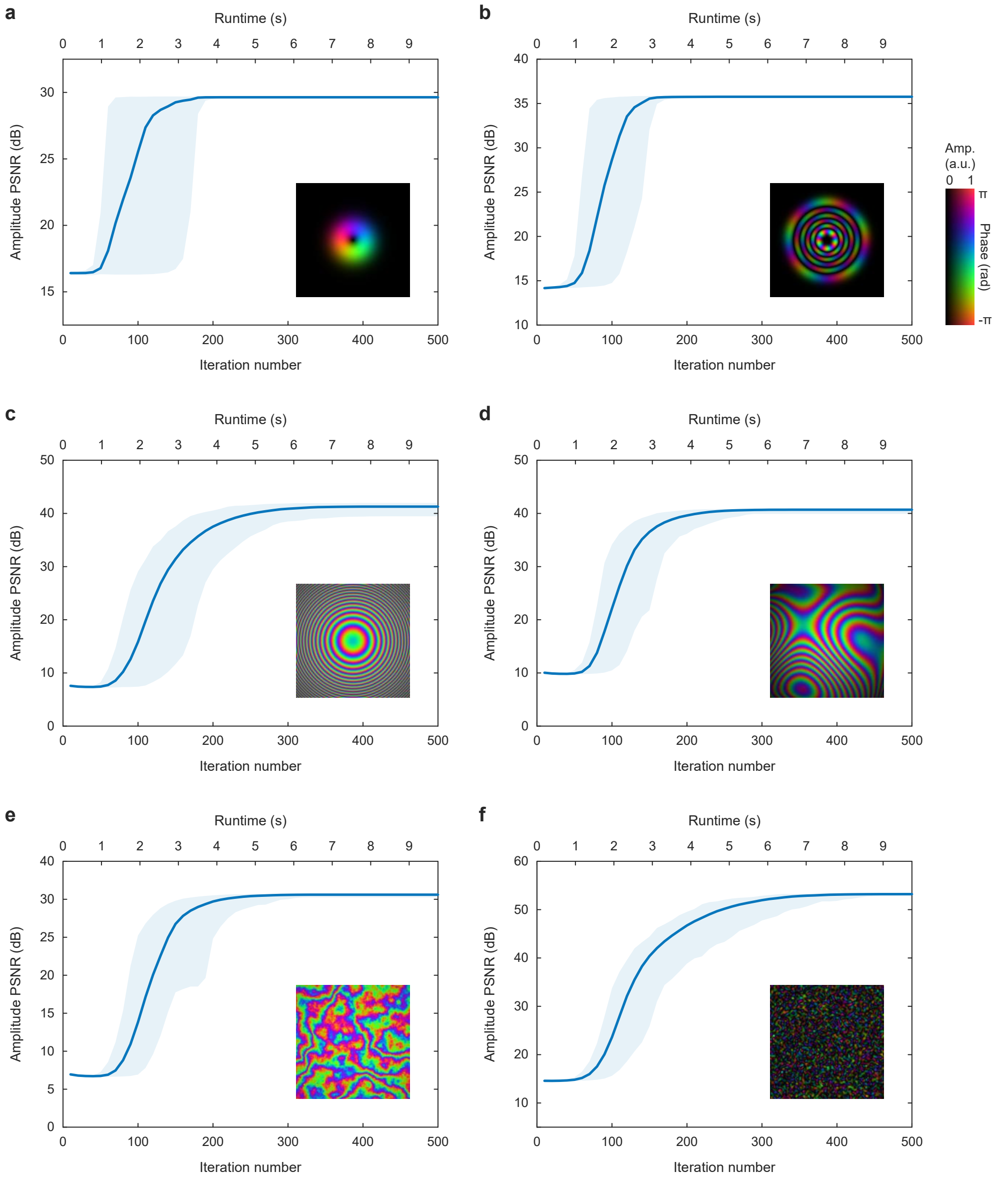

Supplementary Fig. 6 Runtime evaluation of SAFARI based on simulation. The amplitude PSNR value is plotted against iteration number and runtime. The solid line and the shaded region indicate the average and the range obtained from 50 random initializations, respectively. The wavefront profiles are shown in the insets, with a dimension of 1,000×1,000 pixels.

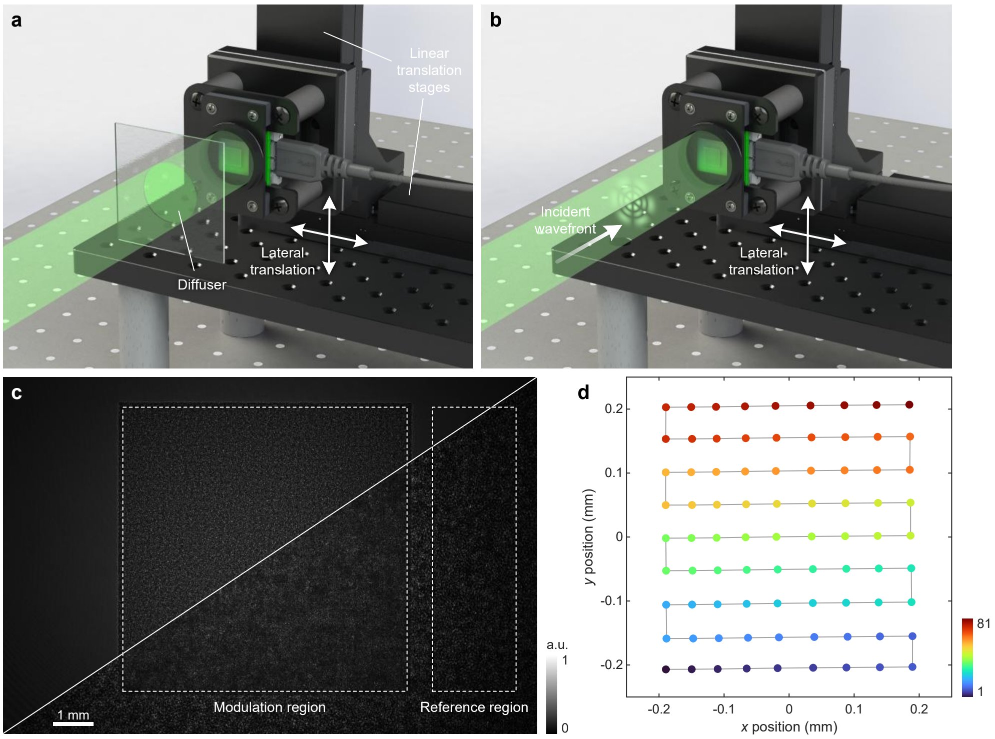

Supplementary Fig. 7 Ptychographic measurement for the DOE transmission function and ground-truth wavefronts. (**a**) Experimental setup for DOE calibration. The wavefront sensor is laterally scanned by a two-dimensional translation stage to acquire ptychographic dataset. A diffuser is placed in the optical path to provide random illumination. (**b**) Experimental setup for ground-truth wavefront measurement. (**c**) Captured raw intensity images under plane wave (upper left) and diffuser (lower right) illumination are shown. The central region corresponds to the DOE modulation region, whereas the surrounding clear region is used for positional tracking. (**d**) Calibrated 9×9 translation positions of the wavefront sensor during ptychographic acquisition.

Supplementary Tables

Supplementary Table 1 Phase reconstruction errors for aberrations and turbulence.

Wavefront	RMS (wavelength)	Description
Fig. 5a	0.0206	Parabolic phase with a focal length of 1000 mm
Fig. 5b	0.0241	Parabolic phase with a focal length of 500 mm
Fig. 5c	0.0337	Parabolic phase with a focal length of 200 mm
Fig. 5d	0.0249	21 Zernike orders, scaling constant $s = 4$
Fig. 5e	0.0310	21 Zernike orders, scaling constant $s = 8$
Fig. 5f	0.0375	21 Zernike orders, scaling constant $s = 12$
Fig. 5g	0.0270	66 Zernike orders, scaling constant $s = 2$
Fig. 5h	0.0313	136 Zernike orders, scaling constant $s = 2$
Fig. 5i	0.0366	231 Zernike orders, scaling constant $s = 2$
Fig. 5j	0.0230	Fourier phase screen with Fried parameter $r_0 = 0.3$ mm
Fig. 5k	0.0328	Fourier phase screen with Fried parameter $r_0 = 0.1$ mm
Fig. 5l	0.0731	Fourier phase screen with Fried parameter $r_0 = 0.03$ mm

Supplementary Notes

1 Mathematical interpretation of SAFARI

The general framework of SAFARI consists of three regularization terms. To study the individual contribution of each regularization term, we performed frequency-domain transfer function analysis based on their proximal operators [1]:

$$\mathrm{prox}_{\gamma R}(\boldsymbol{u}) = \underset{\boldsymbol{x}}{\mathrm{argmin}} \left\{ \frac{1}{2} \|\boldsymbol{x} - \boldsymbol{u}\|_2^2 + \gamma R(\boldsymbol{x}) \right\}. \tag{S1}$$

While in practice the differentiable regularizers R_a and R_c are implemented through gradient updates rather than proximal updates, the similarity between proximal operators and gradient descent updates has been well known [1]:

$$\mathrm{prox}_{\gamma R}(\boldsymbol{u}) \approx \boldsymbol{u} - \gamma \nabla_{\boldsymbol{u}} R(\boldsymbol{u}) \tag{S2}$$

when γ is small and R is differentiable. Thus, from a practical (albeit less rigorous) perspective, apart from enforcing consistency with the forward model, the iterative reconstruction process can be interpreted as sequentially applying proximal updates for each regularization term.

Complex amplitude regularization R_c. The proximal update with respect to the complex amplitude regularization term R_c is given by

$$\mathrm{prox}_{\gamma R_c}(\boldsymbol{u}) = \underset{\boldsymbol{x}}{\mathrm{argmin}} \left\{ \frac{1}{2} \|\boldsymbol{x} - \boldsymbol{u}\|_2^2 + \gamma\lambda_c \|\boldsymbol{D}\boldsymbol{x}\|_2^2 \right\} = \left(\boldsymbol{I} + 2\gamma\lambda_c \boldsymbol{D}^{\mathsf{H}}\boldsymbol{D}\right)^{-1} \boldsymbol{u}. \tag{S3}$$

Note that $\boldsymbol{D}$ consists of two one-dimensional finite-difference operators along the horizontal and vertical directions $\boldsymbol{D} = (\boldsymbol{D}_x^{\mathsf{T}}\, \boldsymbol{D}_y^{\mathsf{T}})^{\mathsf{T}}$. $\boldsymbol{D}_x$ and $\boldsymbol{D}_y$ can also be interpreted as convolution operators with kernels $(1, -1)$ and $(1, -1)^{\mathsf{T}}$, respectively, both of which are diagonalizable in the Fourier domain. Therefore, we have

$$\begin{aligned} \boldsymbol{D}^{\mathsf{H}}\boldsymbol{D} &= \begin{pmatrix} \boldsymbol{D}_{\boldsymbol{x}}^{\mathsf{H}} & \boldsymbol{D}_{\boldsymbol{y}}^{\mathsf{H}} \end{pmatrix} \begin{pmatrix} \boldsymbol{D}_{\boldsymbol{x}} \\ \boldsymbol{D}_{\boldsymbol{y}} \end{pmatrix} = \boldsymbol{D}_x^{\mathsf{H}}\boldsymbol{D}_x + \boldsymbol{D}_y^{\mathsf{H}}\boldsymbol{D}_y \\ &= \boldsymbol{F}^{-1}\mathrm{diag}(|\boldsymbol{h}_x|^2)\boldsymbol{F} + \boldsymbol{F}^{-1}\mathrm{diag}(|\boldsymbol{h}_y|^2)\boldsymbol{F}, \end{aligned} \tag{S4}$$

where $\boldsymbol{h}_x$ and $\boldsymbol{h}_y$ are the transfer functions corresponding to the horizontal and vertical finite difference operators, respectively. Substituting Eq. (S4) into Eq. (S3), we have

$$\mathrm{prox}_{\gamma R_c}(\boldsymbol{u}) = \boldsymbol{F}^{-1}\mathrm{diag}\left(\frac{\boldsymbol{1}}{\boldsymbol{1} + 2\gamma\lambda_c \left(|\boldsymbol{h}_x|^2 + |\boldsymbol{h}_y|^2\right)} \right) \boldsymbol{F}\boldsymbol{u}. \tag{S5}$$

From Eq. (S5), it can be observed that the proximal update of R_c applies a low-pass filtering to the current estimate. The transfer function is shown in Supplementary Note Fig. 1a.

Amplitude regularization R_a. The proximal update with respect to the amplitude regularization term R_a can be similarly derived:

$$\begin{aligned}\text{prox}_{\gamma R_a}(\boldsymbol{u}) &= \underset{\boldsymbol{x}}{\text{argmin}} \left\{ \frac{1}{2} \|\boldsymbol{x} - \boldsymbol{u}\|_2^2 + \gamma\lambda_a \|\boldsymbol{D}|\boldsymbol{x}|\|_2^2 \right\} \\ &= \text{diag}\left(\frac{\boldsymbol{u}}{|\boldsymbol{u}|}\right) \left(\boldsymbol{I} + 2\gamma\lambda_a \boldsymbol{D}^{\mathsf{H}}\boldsymbol{D}\right)^{-1} |\boldsymbol{u}| \\ &= \text{diag}\left(\frac{\boldsymbol{u}}{|\boldsymbol{u}|}\right) \boldsymbol{F}^{-1}\text{diag}\left(\frac{\mathbf{1}}{\mathbf{1} + 2\gamma\lambda_a \left(|\boldsymbol{h}_x|^2 + |\boldsymbol{h}_y|^2\right)}\right) \boldsymbol{F}|\boldsymbol{u}|. \end{aligned} \tag{S6}$$

Equation (S6) implies that $\text{prox}_{\gamma R_a}(\boldsymbol{u})$ has a transfer function identical in form to Eq. (S5), but applies only to the amplitude while leaving the phase unchanged.

Fourier-domain regularization R_f. R_f is an indicator function of set C, whose proximal update corresponds to the projection operator to C:

$$\text{prox}_{\gamma R_f}(\boldsymbol{u}) = \underset{\boldsymbol{x}}{\text{argmin}} \left\{ \frac{1}{2} \|\boldsymbol{x} - \boldsymbol{u}\|_2^2 + \gamma I_C(\boldsymbol{x}) \right\} = \mathcal{P}_C(\boldsymbol{u}) = \boldsymbol{F}^{-1}\text{diag}(\boldsymbol{h}_s)\boldsymbol{F}\boldsymbol{u}, \tag{S7}$$

where the transfer function $\boldsymbol{h}_s$ is a $\{0, 1\}$-valued Boolean function that takes the value 1 inside the support region C and 0 outside. As a result, $\text{prox}_{\gamma R_f}(\boldsymbol{u})$ serves as a brick-wall filter shown in Supplementary Note Fig. 1b.

To briefly summarize, the proximal updates for all three regularization terms serve as low-pass filters but perform in slightly different ways. The spatial-domain regularizers R_a and R_c serve as *soft* low-pass filters that promote lower-frequency components for the amplitude and complex amplitude, respectively, whereas the Fourier-domain regularizer R_f serves as a *hard* low-pass filter that rejects any frequency components larger than $k_{\max}$. We empirically observed that R_a and R_c primarily enhance reconstruction of smoother wavefronts, while R_f proves more effective for complex wavefront features and high-frequency components.

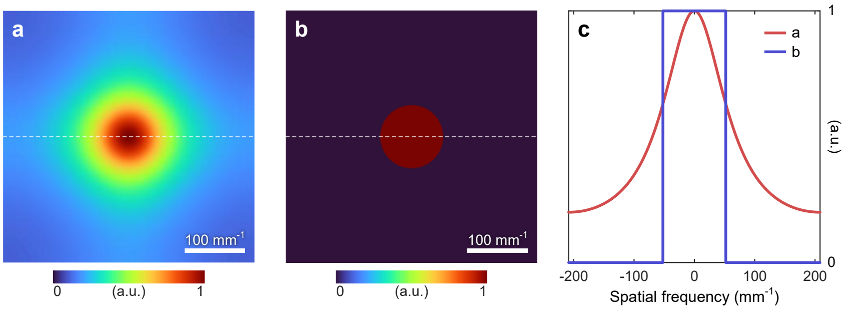

Supplementary Note Fig. 1 Understanding SAFARI from frequency analysis. (**a**) Transfer function of the proximal update with respect to the spatial-domain regularizer R_c with $\lambda_c = 1$ and $\gamma = 1$. (**b**) Transfer function of the proximal update with respect to the Fourier-domain regularizer R_f. (**c**) Cross sections of (**a**) and (**b**).

2 Modeling of the wavefront sensor

The wavefront sensor consists of a DOE that functions as a thin diffuser and an image sensor placed a few millimeters downstream, as illustrated in Supplementary Note Fig. 2. While diffuser-based wavefront sensors have been widely studied in the literature, their working principle often relies on the memory effect, providing phase-gradient measurements. Here, we adopt a different modeling scheme for the imaging process based on scalar diffraction theory, which allows us to directly retrieve the complex amplitude of the wavefront. Below is a step-by-step description of the forward model. A detailed MATLAB implementation can be found in Ref. [2].

(1) Diffuser modulation. The DOE introduces random binary phase modulation to the wavefront, which can be expressed as an element-wise multiplication:

$$U_o = \mathcal{M}(U_i) \overset{\text{def}}{=} M \cdot U_i, \tag{S8}$$

where M denotes the DOE transmission function, and U_i and U_o denote the input and output complex fields, respectively. When converting to vectorized notation, this process can be expressed as an element-wise product with the diffuser transmission function $\boldsymbol{m}$, or equivalently multiplication with a diagonal matrix $\text{diag}(\boldsymbol{m})$.

(2) Free-space propagation. The modulated wavefront then propagates from the DOE plane to the sensor plane, where the free-space propagation can be described based on the angular spectrum model as [3]

$$U_o = \mathcal{Q}(U_i) \overset{\text{def}}{=} \mathcal{F}^{-1}\left\{\mathcal{F}(U_i) \cdot \exp\left[\mathrm{j}\frac{2\pi}{\lambda} d\sqrt{1-(\lambda f_x)^2-(\lambda f_y)^2}\right]\right\}, \tag{S9}$$

where λ denotes wavelength, f_x and f_y denote the spatial frequency coordinates, j is the imaginary unit, and $\mathcal{F}$ and $\mathcal{F}^{-1}$ denote the Fourier transform and its inverse, respectively. This process can be expressed as an abstract matrix operator $\boldsymbol{Q}$ in the vectorized notation.

(3) Sensor response. The image sensor only responds to the intensity (or equivalently, amplitude) of the incident wavefront. Therefore, the captured amplitude image corresponds the modulus of the incident wavefront:

$$U_o = |U_i|. \tag{S10}$$

Taking the above steps together, we arrive at the forward model of the wavefront sensor:

$$Y = |\mathcal{Q} \circ \mathcal{M}(X)|, \tag{S11}$$

where X and Y denote the complex amplitude of the wavefront and the raw measurement, respectively. The vectorized form of Eq. (S11) is

$$\boldsymbol{y} = |\boldsymbol{Q}\text{diag}(\boldsymbol{m})\boldsymbol{x}|. \tag{S12}$$

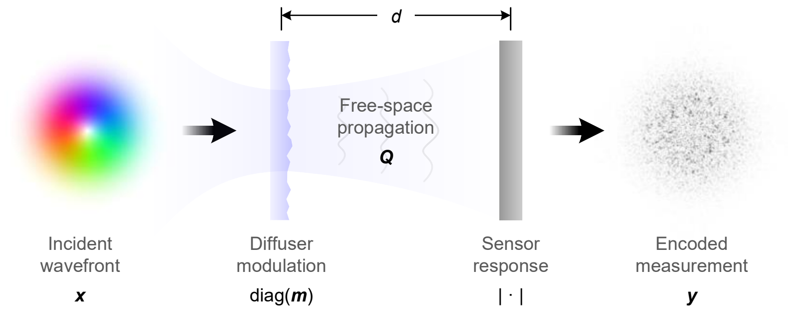

Supplementary Note Fig. 2 Forward model of the wavefront sensor. The physical processes and the corresponding mathematical operators are shown.

3 Quantitative evaluation of SAFARI

In this section, we conduct simulation studies to quantitatively evaluate SAFARI's performance and validate its effectiveness across diverse wavefront sensing tasks. All simulation parameters, including pixel size, wavelength, DOE feature size, DOE phase retardance, and sensor noise level, are kept as consistent as possible with the experimental conditions, unless otherwise specified.

3.1 Robustness of the physical priors

The introduction of a hard support constraint in the Fourier domain may raise practical concerns regarding its generalizability to different wavefronts. To investigate the robustness of such physical prior knowledge, we conducted simulation studies on reconstructing wavefronts with varying Fourier spectrum distributions. Supplementary Note Figure 3 shows six representative cases of speckle field reconstruction with arbitrarily shaped Fourier spectra. These speckle fields were generated by assigning specified amplitude profiles and random phases uniformly sampled in $[0, 2\pi)$ to their Fourier spectra. Their Fourier spectra differ in size, shape, and amplitude profile, and the resulting spatial statistics also vary across cases. Nevertheless, the wavefront is accurately retrieved in all cases, as indicated by the uniform phase profiles of the phase-conjugated wavefronts. These results demonstrate that SAFARI does not impose stringent requirements on the Fourier spectrum of the wavefront. Notably, when the wavefront spectrum extends beyond the support region, the reconstructed wavefront deviates slightly from the ground truth due to missing spectral components, yet the low-frequency content within the support region is still well estimated.

In addition to speckles, we examined SAFARI's tolerance to spectrum mismatch by reconstructing synthetic wavefronts with amplitude and phase maps drawn from natural images from the DIV2K dataset [4], as shown in Supplementary Note Fig. 4. For both amplitude and phase objects, the band-limited wavefronts can be accurately reconstructed. This is largely because most natural scenes have their energy concentrated in the low-frequency regime, and SAFARI is expected to fail when high-frequency components beyond the cutoff frequency dominate.

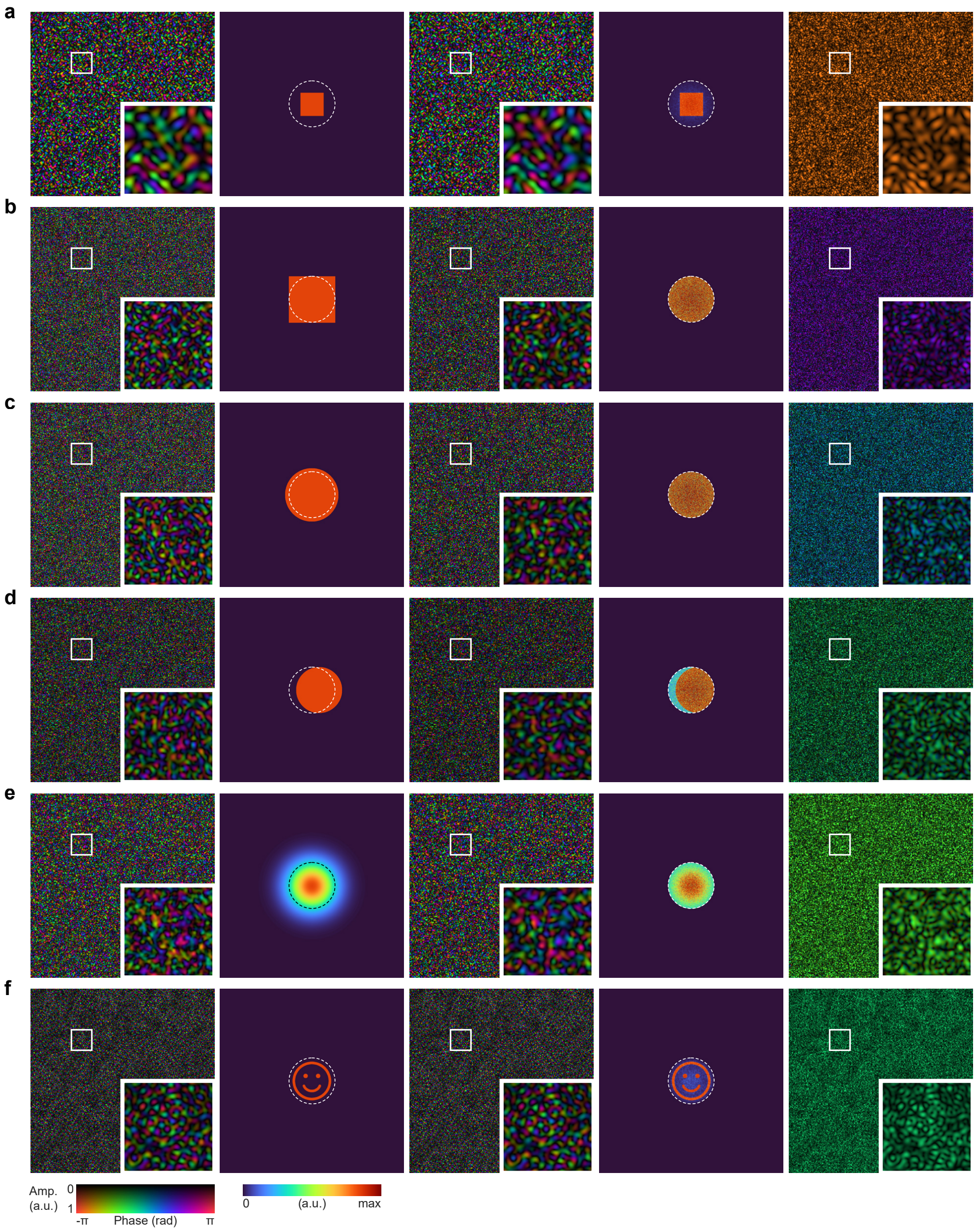

Supplementary Note Fig. 3 Simulation study on SAFARI's robustness in retrieving speckles with different Fourier magnitudes. From left to right: complex amplitude of the ground-truth wavefront $\boldsymbol{x}_{\mathrm{ref}}$, Fourier magnitude of $\boldsymbol{x}_{\mathrm{ref}}$, complex amplitude of the reconstructed wavefront $\hat{\boldsymbol{x}}$, Fourier magnitude of $\hat{\boldsymbol{x}}$, and phase-conjugated wavefront $\hat{\boldsymbol{x}} \odot \exp(-\mathrm{j} \arg \boldsymbol{x}_{\mathrm{ref}})$. The insets show the enlarged views, and the dashed circle indicates the Fourier support region.

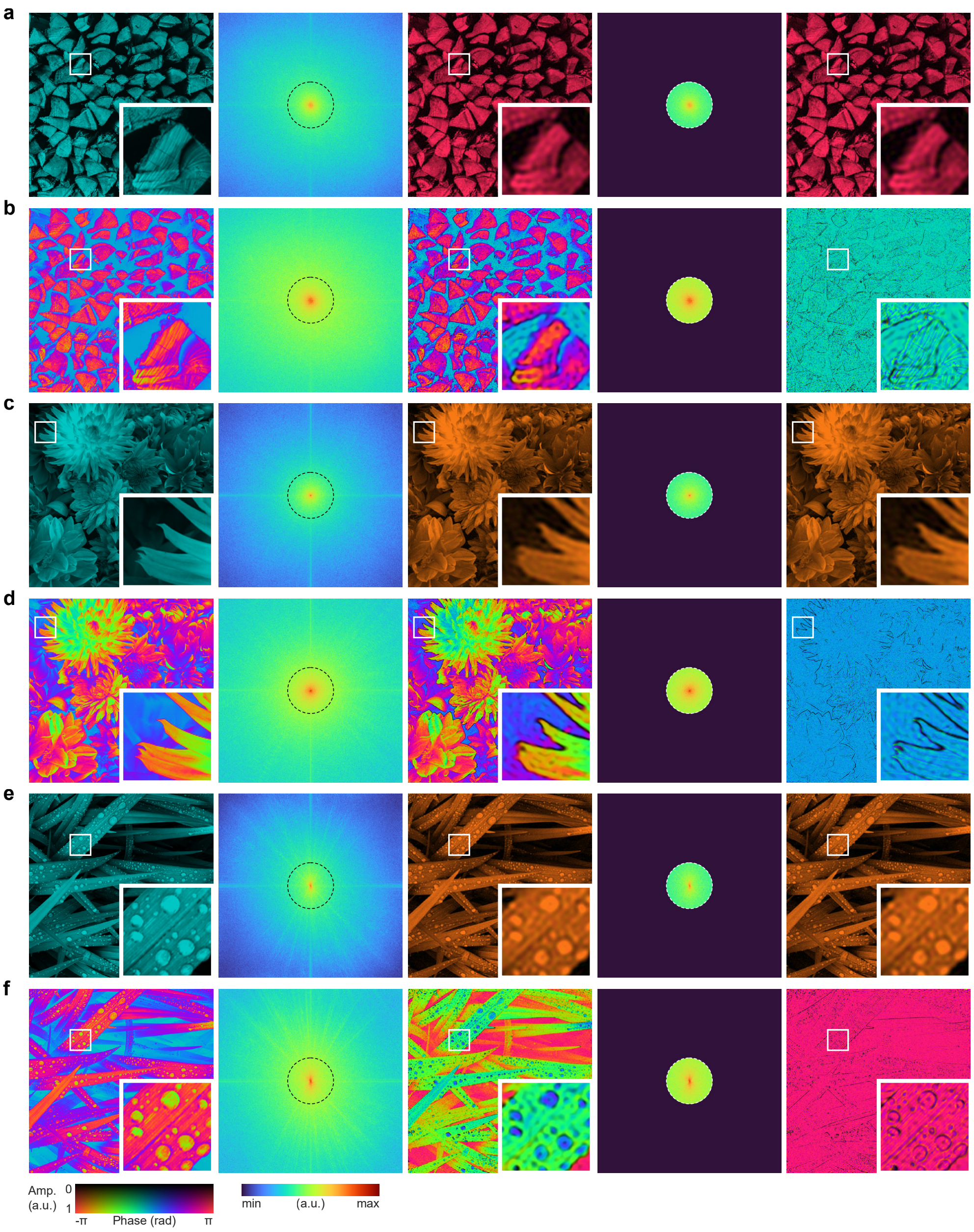

Supplementary Note Fig. 4 Simulation study on SAFARI's robustness in retrieving synthetic amplitude and phase objects. From left to right: complex amplitude of the ground-truth wavefront $\boldsymbol{x}_{\mathrm{ref}}$, Fourier magnitude of $\boldsymbol{x}_{\mathrm{ref}}$, complex amplitude of the reconstructed wavefront $\hat{\boldsymbol{x}}$, Fourier magnitude of $\hat{\boldsymbol{x}}$, and phase-conjugated wavefront $\hat{\boldsymbol{x}} \odot \exp(-\mathrm{j} \arg \boldsymbol{x}_{\mathrm{ref}})$. The Fourier magnitudes are shown in a logarithmic scale for better visibility. The insets show the enlarged views, and the dashed circle indicates the Fourier support region.

3.2 Parameter tuning

To evaluate SAFARI's dependence on parameter tuning, we reconstructed diverse simulated wavefronts using a fixed set of algorithm parameters, as shown in Supplementary Note Fig. 5. We set $\lambda_a = \lambda_c = 1 \times 10^{-2}$ and the Fourier support radius to 1/8 of the sensor bandwidth. With this configuration, SAFARI successfully reconstructed wavefronts across extreme cases with markedly different spatial features and Fourier statistics, including Laguerre-Gaussian beams with azimuthal orders of 1 and 150 (Supplementary Note Fig. 5a, b), Hermite-Gaussian beams with mode indices of (1,0) and (30,30) (Supplementary Note Fig. 5c, d), parabolic phases with focal lengths of 1000 mm and 80 mm (Supplementary Note Fig. 5e, f), speckle fields with different mode densities (Supplementary Note Fig. 5g-i), Zernike aberrations with varying numbers of modes (Supplementary Note Fig. 5j-l), other structured beams or turbulent wavefronts (Supplementary Note Fig. 5m-o), an amplitude image (Supplementary Note Fig. 5p), as well as challenging cases approaching the dynamic range and spatial resolution limits (Supplementary Note Fig. 5q, r). These results indicate that SAFARI is relatively insensitive to the choice of regularization parameters, and a single set of fixed parameters is suitable for most application scenarios.

In the experiments, considering various sources of error and noise, the optimal regularization parameters λ_a and λ_c are typically tuned between 1×10^{-3} and 1×10^{-1}, depending on the expected smoothness of the wavefront.

3.3 Stagnation issue and initialization strategy

The underlying inverse problem in SAFARI is a nonconvex optimization task. Consequently, the optimizer may stagnate at local minima, degrading wavefront estimation. We empirically observed that stagnation is often associated with phase vortices in the reconstructed wavefront, as illustrated in Supplementary Note Fig. 6. Artifacts also appear in the amplitude channel around the branch point. The data-fidelity loss $F(\boldsymbol{x})$ in Eq. (2) is typically larger when stagnating at suboptimal points, which can be used to identify such cases. To address this issue, a practical approach is to adopt a multi-start strategy, where the algorithm is run with multiple random initializations and the solution with the lowest data-fidelity loss is selected. This provides a viable workaround, albeit at the cost of longer runtimes.

Additionally, the reconstruction time can be further shortened if certain prior knowledge of the wavefront is available. For example, estimating the global speckle displacement can reveal the global linear phase shift (i.e., tip and tilt) of the wavefront. Incorporating prior knowledge of low-order aberrations into the algorithm initialization can significantly accelerate convergence and reduce the required number of iterations, particularly when reconstructing highly tilted wavefronts.

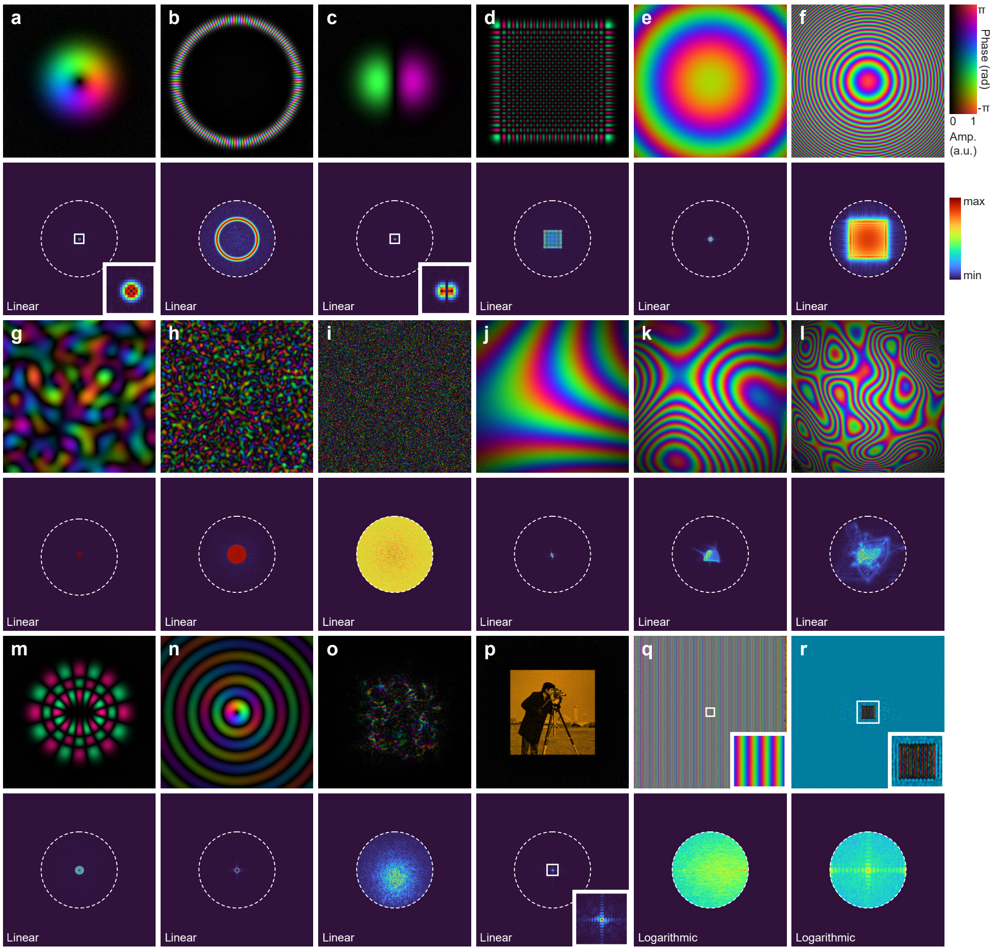

Supplementary Note Fig. 5 Simulated reconstruction of diverse wavefronts with fixed algorithm parameters. For each wavefront, the reconstructed complex amplitude and Fourier magnitude are shown. The dashed circle indicates the support region. For better visibility, some of the Fourier magnitudes are shown in linear scale while some others are shown in logarithmic scale.

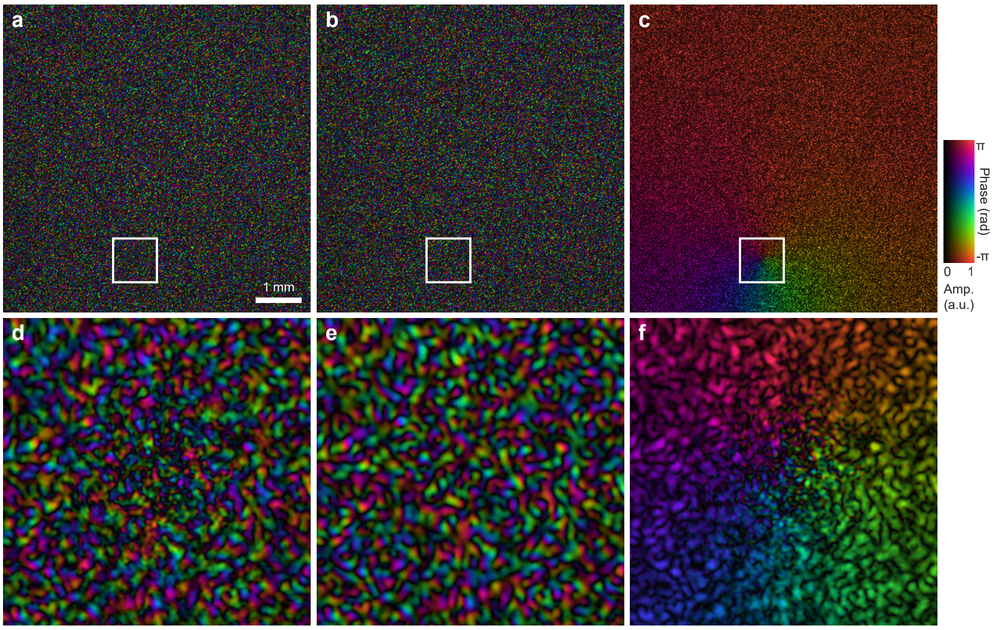

Supplementary Note Fig. 6 Failure case of speckle field reconstruction. Complex amplitudes of (**a**) the reconstructed wavefront $\hat{\boldsymbol{x}}$, (**b**) the reference wavefront $\boldsymbol{x}_{\mathrm{ref}}$, and (**c**) the phase-conjugated wavefront $\hat{\boldsymbol{x}} \odot \exp(-\mathrm{j} \arg \boldsymbol{x}_{\mathrm{ref}})$ are shown. (**d-f**) Enlarged views of the boxed regions in (**a-c**). A vortex phase can be seen from the phase-conjugated wavefront.

3.4 Generalizability to other types of wavefront

Due to the experimental challenges of obtaining reproducible atmospheric turbulence, we evaluated SAFARI's generalizability to turbulent wavefronts through simulation. Synthetic turbulent wavefronts were generated using the layered atmospheric model [5], with the turbulence strength controlled by varying the number of phase screens through which the source propagated. Supplementary Note Figure 7a-d presents four representative reconstructions corresponding to 3, 5, 10, and 20 phase screens, respectively. Compared with the single-layer phase screen experimentally demonstrated with a phase-only SLM, these simulated wavefronts exhibit more realistic spatial profiles with strong scintillation, particularly as the number of phase screens increases. In all four cases, the turbulent wavefronts are accurately retrieved, although slight phase residuals are observed for ultra-strong turbulence due to the Fourier spectrum cutoff, as shown in Supplementary Note Fig. 7d.

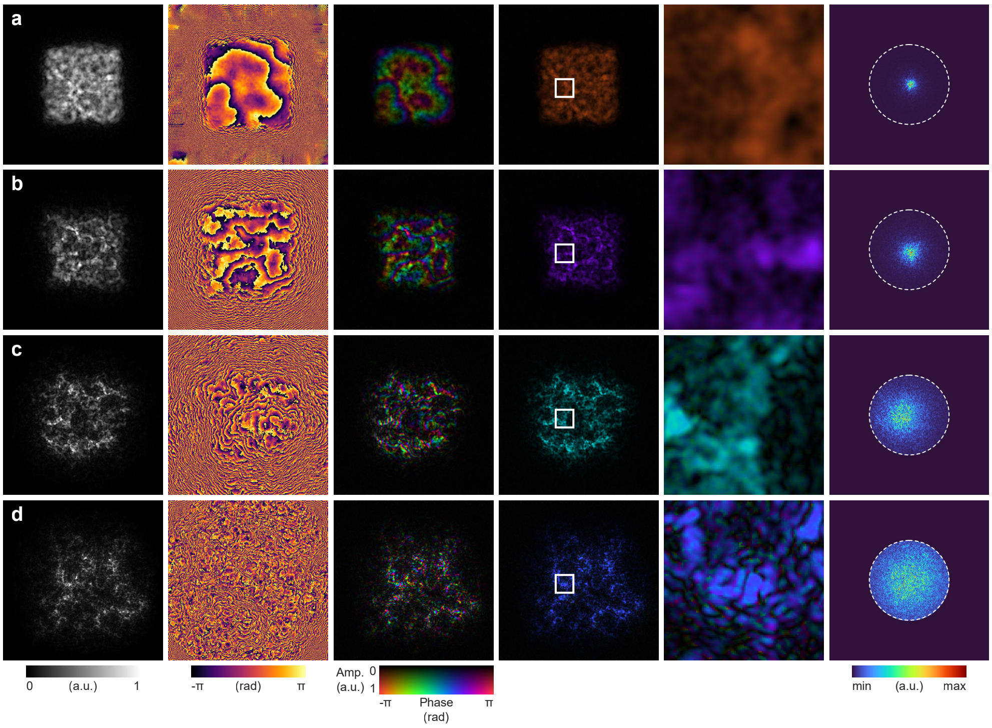

Supplementary Note Fig. 7 Simulation study of reconstructing atmospheric turbulence. The numbers of phase screens used to generate the wavefronts are 3 (**a**), 5 (**b**), 10 (**c**), and 20 (**d**), respectively. From left to right: amplitude and phase profiles of the synthetic wavefront $\boldsymbol{x}_{\mathrm{ref}}$, reconstructed complex amplitude $\hat{\boldsymbol{x}}$, phase-conjugated wavefront $\hat{\boldsymbol{x}} \odot \exp(-\mathrm{j} \arg \boldsymbol{x}_{\mathrm{ref}})$, enlarged view of the region of interest, and enlarged Fourier magnitude of $\hat{\boldsymbol{x}}$.

3.5 Extending SAFARI to generalized SHWFSs

In principle, SAFARI is a wavefront reconstruction framework that is not restricted to specific measurement schemes. We present a simple simulation study on three popular sensor configurations for generalized SHWFSs, including the microlens array-based classical SHWFS, the cross grating-based quadriwave lateral shearing interferometer (QWLSI), and the diffuser-based wavefront sensor. The simulation parameters are summarized in Supplementary Note Table 1. As shown in Supplementary Note Fig. 8a-c, while these configurations produce markedly different field amplitude profiles, conventional reconstruction methods apply similar flow-tracking procedures to derive the phase gradient. Here, SAFARI is adapted to the specific forward models and employed as an alternative reconstruction approach, capable of directly retrieving the complex amplitude of the wavefront.

We synthesized 50 random speckle fields and performed reconstruction from simulated measurements. Considering the global phase ambiguity and the existence of phase singularities, direct comparison of the reconstructed complex amplitude may not genuinely reflect the actual reconstruction quality. After obtaining the reconstructed wavefront $\hat{\boldsymbol{x}}$, we apply a virtual phase conjugation to the speckle field as $\boldsymbol{x}_{\mathrm{ref}} \odot \exp(-\mathrm{j} \arg \hat{\boldsymbol{x}})$ and focus the corrected wavefront using a virtual lens. An ideally corrected wavefront should have a uniform phase profile and a bright focal spot. Therefore, we introduce relative focusing efficiency (RFE) as the quality metric, which is defined as the ratio between the total energy distribution within the main lobe of the focal spots from the phase-conjugated fields using $\hat{\boldsymbol{x}}$ (i.e., $\boldsymbol{x}_{\mathrm{ref}} \odot \exp(-\mathrm{j} \arg \hat{\boldsymbol{x}})$) and the ideal wavefront $\boldsymbol{x}_{\mathrm{ref}}$ (i.e., $\boldsymbol{x}_{\mathrm{ref}} \odot \exp(-\mathrm{j} \arg \boldsymbol{x}_{\mathrm{ref}})$). Supplementary Note Figure 8d-f shows the calculated RFEs at different DOE-to-sensor distances for the three measurement schemes. It can be observed that all three configurations benefit from a larger distance, because stronger interference between distant points of the incident wavefront occurs as the propagation distance increases. Notably, the microlens array-based SHWFS demonstrates improved performance at defocused positions, which differs from its conventional usage. This can also be explained by the reduced interference and consequently increased ambiguity between neighboring sub-apertures at the focal plane. While SAFARI achieves the best reconstruction quality for diffuser-based wavefront sensors, complex wavefront reconstruction is made possible in other configurations as well. We anticipate that incorporating model-specific modifications, such as combining SAFARI with classical flow-tracking algorithms for SHWFS or spectrum-filtering algorithms for QWLSI, could further enhance performance.

Supplementary Note Table 1 Simulation parameters.

**Diffuser-based WFS**		**QWLSI**		**SHWFS**	
Pixel size	5 μm	Pixel size	5 μm	Pixel size	5 μm
Wavelength	500 nm	Wavelength	500 nm	Wavelength	500 nm
Diffuser feature size	25 μm	Grating period	40 μm	Lens diameter	160 μm

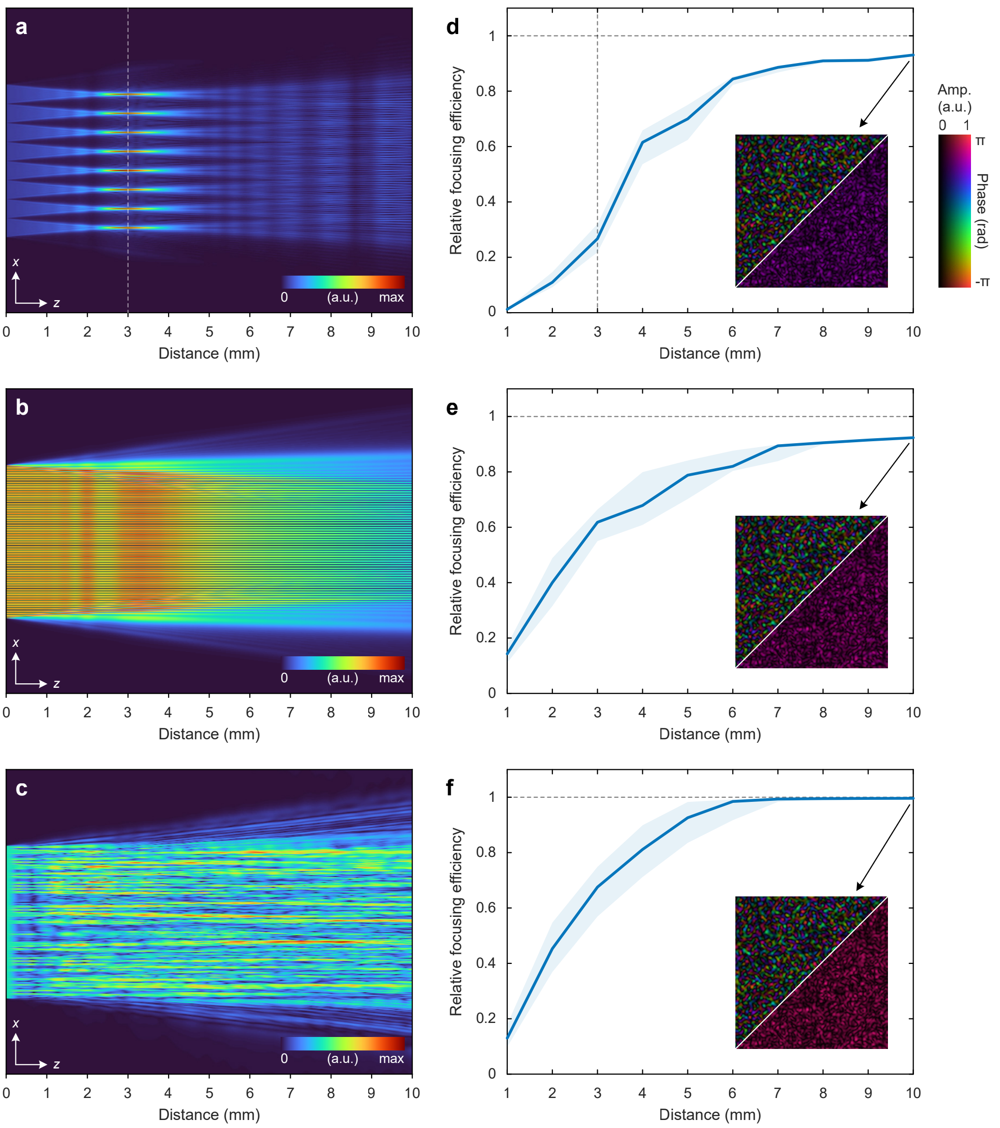

Supplementary Note Fig. 8 Simulation study of extending SAFARI to generalized SHWFSs. (**a-c**) $x-z$ cross-sectional amplitude profiles of the diffracted field under plane wave illumination for three typical measurement schemes: microlens array (**a**), cross grating (**b**), and diffuser (**c**). (**d-f**) Wavefront reconstruction performance for different DOE-to-sensor distances corresponding to (**a**), (**b**), and (**c**), respectively. The calculated relative focusing efficiency after phase conjugation is used as the quality metric. The solid line and the shaded region indicate the median and the interquartile range across 50 random speckle wavefronts. Insets display the reconstructed complex amplitude (upper left) and the phase-conjugated profile (lower right) for a representative test case. The focal length of the microlenses is 3 mm and is highlighted in (**a**) and (**d**).

4 Comparison with existing wavefront sensing methods

Supplementary Note Table 2 Comparison with commercial wavefront sensors.

Method	Product	Wavefront dim.	Spatial resolution	Dynamic range	Accuracy (RMS)
SHWFS	Thorlabs WFS31-7AR	73×45	150 μm	$500\lambda^{a}$	$\lambda/40$ / 15.8 nm[a]
SHWFS	Thorlabs WFS31-14AR	35×21	300 μm	$250\lambda^{a}$	$\lambda/60$ / 10.6 nm[a]
QWLSI	Phasics SID4 HR	416×360	24 μm	500 μm	20 nm
This work		700×700	9.6 μm	313λ / 166 μm[b]	$\lambda/30$ / 17.6 nm[b]

[a] Evaluated at 633 nm.
[b] Evaluated at 532 nm.

Supplementary Note Table 3 Comparison with existing wavefront sensing methods for speckle field characterization. CMI, coherent modulation imaging. SSM, speckle-correlation scattering matrix. MMF, multimode fiber.

Method	# modes	Wavefront dim.[a]	Application	Refs.
CMI	10-50[b]	–	General wavefronts	[6, 7]
Diffuser-based	133	20,000	General wavefronts	[8, 9]
QWLSI	≈ 3,000	≈ 683×683[c]	General wavefronts	[10]
Image-based	5,796	N.A.[d]	Speckles from MMFs	[11]
SSM	22,500[e]	150×150	General wavefronts	[12]
This work	≈ 190,000	700×700	General wavefronts	

[a] Calculated by dividing the FOV by the half-pitch resolution, or according to the number of effective phase sampling points.
[b] Only OAM modes are measured in Ref. [6]. The maximum number of detectable spatial modes could be potentially increased by considering multiple degrees of freedom.
[c] Estimated assuming the optimal 1/3 bandwidth utilization according to Ref. [13].
[d] The mode coefficients, rather than the spatial profile, are retrieved.
[e] Calculated theoretically according to the dimension of the transmission matrix.

5 Calibration of the spatial light modulator

The SLM takes an 8-bit grayscale pattern as input and produces a corresponding pixel-wise phase retardance. The relationship between the input grayscale and the corresponding phase retardance is calibrated in advance to synthesize ideal wavefronts. Supplementary Note Figure 9a shows the experimental setup widely adopted for SLM phase response calibration [14]. In the illumination path, the polarizer (P1) and the half-wave plate are adjusted such that the transmitted light has a polarization angle of 45° with respect to the orientation axis of the SLM. In the detection path, the analyzer (P2) is rotated to -45° and the filtered intensity is captured. During calibration, uniform grayscale patterns between 0 and 255 are sequentially addressed to the SLM, and the corresponding output light intensity is measured. The intensity signal follows the relationship $I \propto 1 - \cos \Delta\phi$, where $\Delta\phi$ denotes the phase retardance between the x- and y-polarized light. Supplementary Note Figure 9b plots the measured intensity versus input grayscale, showing good agreement with theoretical expectations.

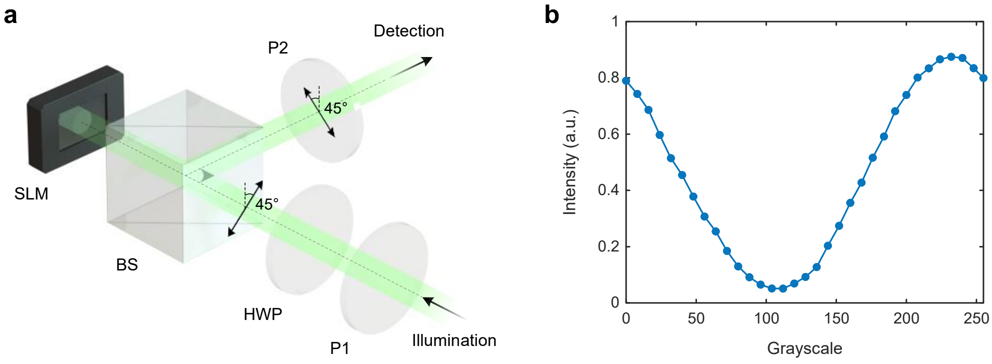

Supplementary Note Fig. 9 Phase response characterization of the SLM. (**a**) Experimental setup. Polarizer P1 and HWP are adjusted to produce 45° linear polarization relative to the SLM orientation axis, and the -45° polarization state is selected in the output path for intensity detection. (**b**) Measured light intensity as a function of the SLM grayscale value.

6 Algorithm derivation and convergence theory

6.1 Derivation of the proximal gradient algorithm

As described in the main text, the wavefront reconstruction algorithm for SAFARI is based on the proximal gradient method (Eqs. (7)-(9)). While straightforward, a brief derivation of the algorithm is presented here for completeness. The differentiable terms $F(\boldsymbol{x})$, $R_a(\boldsymbol{x})$, and $R_c(\boldsymbol{x})$ are minimized using a gradient descent step given by Eq. (7). The Wirtinger gradients are calculated as

$$\nabla_{\boldsymbol{x}} F = \boldsymbol{A}^{\mathsf{H}} \mathrm{diag}\left(\frac{\boldsymbol{A}\boldsymbol{x}}{|\boldsymbol{A}\boldsymbol{x}|}\right)(|\boldsymbol{A}\boldsymbol{x}| - \boldsymbol{y}), \tag{S13}$$

$$\nabla_{\boldsymbol{x}} R_a = \lambda_a \text{diag}\left(\frac{\boldsymbol{x}}{|\boldsymbol{x}|}\right) \boldsymbol{D}^{\mathsf{H}} \boldsymbol{D} |\boldsymbol{x}|, \tag{S14}$$

$$\nabla_{\boldsymbol{x}} R_c = \lambda_c \boldsymbol{D}^{\mathsf{H}} \boldsymbol{D} \boldsymbol{x}, \tag{S15}$$

where $(\cdot)^{\mathsf{H}}$ denotes the conjugate transpose. The proximal update with respect to the non-differentiable term $R_f(\boldsymbol{x})$ corresponds to the projection operator onto set C. One can easily prove that this projection can be explicitly expressed as

$$\text{prox}_{\gamma R_f}(\boldsymbol{x}) = \mathcal{P}_C(\boldsymbol{x}) = \boldsymbol{F}^{-1} \text{diag}(\boldsymbol{h}_s) \boldsymbol{F} \boldsymbol{x}. \tag{S16}$$

Equation (S16) implies that the proximal update with respect to R_f is simply applying a brick-wall low-pass filter with a cutoff frequency of $k_{\max}$.

To ensure stable convergence, the step size γ for the algorithm is selected as

$$\gamma \leq \frac{1}{L}, \tag{S17}$$

where $L = L_F + L_{R_a} + L_{R_c}$ is the Lipschitz bound of the differentiable terms. In the following section, we derive the analytical bounds for the three terms, which are summarized below:

$$L_F = \rho(\boldsymbol{A}^{\mathsf{H}} \boldsymbol{A}), \tag{S18}$$

$$L_{R_a} = \lambda_a \rho(\boldsymbol{D}^{\mathsf{H}} \boldsymbol{D}), \tag{S19}$$

$$L_{R_c} = \lambda_c \rho(\boldsymbol{D}^{\mathsf{H}} \boldsymbol{D}), \tag{S20}$$

where $\rho(\cdot)$ denotes the spectral radius.

6.2 Proof of convergence

In this Section, we present a theoretical analysis of the Lipschitz bound for the objective function of SAFARI (Eq. (2) in the main text), thereby providing practical guidelines for step size selection with stable convergence.

6.2.1 Preliminaries

Since we are primarily dealing with real-valued functions over complex-valued variables, we adopt the CR-calculus as a helpful mathematical tool for analysis. The CR-calculus extends the complex derivative to general non-analytic functions. Readers may refer to Ref. [15] for a detailed introduction. The CR-calculus regards the complex variable $\boldsymbol{x}$ and its conjugate $\bar{\boldsymbol{x}}$ as independent variables. For example, the fidelity function $F(\boldsymbol{x})$ should be interpreted as a function over the pair of conjugate vectors $\tilde{\boldsymbol{x}} = (\boldsymbol{x}^{\mathsf{T}}, \bar{\boldsymbol{x}}^{\mathsf{T}})^{\mathsf{T}} \in \mathbb{C}^{2n}$, where n is the dimension of $\boldsymbol{x}$. Nevertheless, to keep the notation consistent, we still denote the function as $F(\boldsymbol{x})$. The same applies to other functions as well.

The followings are some intermediate results from matrix analysis, which are helpful for proving the convergence theorem.

Lemma 1. *Given matrices* $\boldsymbol{P} \in \mathbb{C}^{n\times n}$, $\boldsymbol{Q} \in \mathbb{C}^{n\times n}$, *and* $\boldsymbol{R} \in \mathbb{C}^{n\times n}$, *the following holds* [16]*:*
(i) $\boldsymbol{P} \succeq \boldsymbol{Q} \succ \mathbf{0} \Rightarrow \boldsymbol{Q}^{-1} \succeq \boldsymbol{P}^{-1} \succ \mathbf{0}$,
(ii) $\boldsymbol{P} \succ \boldsymbol{Q} \Rightarrow \boldsymbol{R}^{\mathsf{H}}\boldsymbol{P}\boldsymbol{R} \succ \boldsymbol{R}^{\mathsf{H}}\boldsymbol{Q}\boldsymbol{R}$.

Lemma 2. *Given two symmetric matrices* $\boldsymbol{P} \in \mathbb{R}^{n\times n}$ *and* $\boldsymbol{Q} \in \mathbb{R}^{n\times n}$ *and assuming that* $\boldsymbol{P} \succeq \boldsymbol{Q} \succ \mathbf{0}$, *the following holds:*

$$\boldsymbol{Q}^{\mathsf{T}}\boldsymbol{P}^{-1}\boldsymbol{Q} \preceq \boldsymbol{P}. \tag{S21}$$

Proof. According to Lemma 1(i), $\boldsymbol{Q}^{-1} \succeq \boldsymbol{P}^{-1} \succ \mathbf{0}$. Then, we have

$$\boldsymbol{Q}^{\mathsf{T}}\boldsymbol{P}^{-1}\boldsymbol{Q} \overset{(a)}{\preceq} \boldsymbol{Q}^{\mathsf{T}}\boldsymbol{Q}^{-1}\boldsymbol{Q} = \boldsymbol{Q}^{\mathsf{T}} = \boldsymbol{Q} \preceq \boldsymbol{P}, \tag{S22}$$

where (a) is based on Lemma 1(ii). □

Lemma 3 (Schur Complement [16])**.** *Given a* $2n \times 2n$ *Hermitian matrix*

$$\boldsymbol{P} = \begin{pmatrix} \boldsymbol{P}_{11} & \boldsymbol{P}_{12} \\ \boldsymbol{P}_{21} & \boldsymbol{P}_{22} \end{pmatrix}, \tag{S23}$$

where each block is of size $n \times n$, *and we have* $\boldsymbol{P}_{11}^{\mathsf{H}} = \boldsymbol{P}_{11}$, $\boldsymbol{P}_{22}^{\mathsf{H}} = \boldsymbol{P}_{22}$, *and* $\boldsymbol{P}_{12}^{\mathsf{H}} = \boldsymbol{P}_{21}$. *Then*

$$\boldsymbol{P} \succ \mathbf{0} \Leftrightarrow \boldsymbol{P}_{11} \succ \mathbf{0} \quad \text{and} \quad \boldsymbol{P}_{22} - \boldsymbol{P}_{21}\boldsymbol{P}_{11}^{-1}\boldsymbol{P}_{12} \succ \mathbf{0}. \tag{S24}$$

Lemma 4. *Let* $\boldsymbol{D} \in \mathbb{R}^{2n\times n}$ *be the gradient operator for two-dimensional signals* $\boldsymbol{x} \in \mathbb{C}^n$, *the following holds:*

$$\rho(\boldsymbol{D}^{\mathsf{T}}\boldsymbol{D}) \cdot \mathbf{1} \geq \frac{\boldsymbol{D}^{\mathsf{T}}\boldsymbol{D}|\boldsymbol{x}|}{|\boldsymbol{x}|}, \quad \forall \boldsymbol{x} \in \mathbb{C}^n, \tag{S25}$$

where $\mathbf{1} \in \mathbb{R}^n$ *denotes the all-ones vector.*

Proof. We only need to prove that

$$\rho(\boldsymbol{D}^{\mathsf{T}}\boldsymbol{D}) \cdot \mathbf{1} \overset{(1)}{\geq} 4 \cdot \mathbf{1} \overset{(2)}{\geq} \frac{\boldsymbol{D}^{\mathsf{T}}\boldsymbol{D}|\boldsymbol{x}|}{|\boldsymbol{x}|}. \tag{S26}$$

(1) We only need to construct a vector $\boldsymbol{x} \in \mathbb{C}^n$ such that $\|\boldsymbol{D}\boldsymbol{x}\|_2^2 \geq 4\|\boldsymbol{x}\|_2^2$. Let $X_{i,j} = (-1)^{i+j}$ for all $1 \leq i \leq n_x, 1 \leq j \leq n_y$, where n_x and n_y are the dimensions of $\boldsymbol{X}$ and satisfies $n = n_x n_y$. Then, we have

$$\begin{aligned} \|\boldsymbol{D}\boldsymbol{x}\|_2^2 &= 4(n_x - 1)n_y + 4n_x(n_y - 1) = 8n_xn_y - 4n_x - 4n_y \\ &= 4(n_x - 1)(n_y - 1) - 4 + 4n_xn_y \overset{(c)}{\geq} 4n_xn_y = 4\|\boldsymbol{x}\|_x^2, \end{aligned} \tag{S27}$$

where (c) holds for all $n_x, n_y \geq 2$.

(2) Let $\boldsymbol{u} = \boldsymbol{D}^\mathsf{T}\boldsymbol{D}|\boldsymbol{x}|$. According to the definition of $\boldsymbol{D}$, one can verify that

$$U_{i,j} = 4|X_{i,j}| - |X_{i+1,j}| - |X_{i-1,j}| - |X_{i,j+1}| - |X_{i,j-1}| \leq 4|X_{i,j}| \tag{S28}$$

for all $1 \leq i \leq n_x, 1 \leq j \leq n_y$, where the replicate boundary condition is used. Therefore, we have

$$\boldsymbol{D}^\mathsf{T}\boldsymbol{D}|\boldsymbol{x}| \geq 4|\boldsymbol{x}|, \quad \forall \boldsymbol{x} \in \mathbb{C}^n. \tag{S29}$$

Combining Eqs. (S27) and (S29), we arrive at Eq. (S26) and thus complete the proof. □

6.2.2 Convergence of the algorithm

Recall that the wavefront reconstruction is recast as a optimization problem in the following form:

$$\min_{\boldsymbol{x}} \left\{ \underbrace{\||\boldsymbol{A}\boldsymbol{x}| - \boldsymbol{y}\|_2^2}_{F(\boldsymbol{x})} + \underbrace{\lambda_a \|\boldsymbol{D}|\boldsymbol{x}|\|_2^2}_{R_a(\boldsymbol{x})} + \underbrace{\lambda_c \|\boldsymbol{D}\boldsymbol{x}\|_2^2}_{R_c(\boldsymbol{x})} + \underbrace{I_C(\boldsymbol{x})}_{R_f(\boldsymbol{x})} \right\}. \tag{S30}$$

To prove that the proximal gradient algorithm converges with a step size of γ, it is sufficient to show that given any $\boldsymbol{z} \in \mathbb{C}^n$, the differentiable part of the objective function $J(\boldsymbol{x}) \equiv F(\boldsymbol{x}) + R_a(\boldsymbol{x}) + R_c(\boldsymbol{x})$ is upper-bounded by a quadratic function $Q(\boldsymbol{x})$ defined as follows:

$$J(\boldsymbol{x}) \leq Q(\boldsymbol{x}) \stackrel{\text{def}}{=} J(\boldsymbol{z}) + \langle \nabla J(\boldsymbol{z}), \tilde{\boldsymbol{x}} - \tilde{\boldsymbol{z}} \rangle + \frac{L}{2} \|\tilde{\boldsymbol{x}} - \tilde{\boldsymbol{z}}\|_2^2, \quad \forall \boldsymbol{x} \in \mathbb{C}^n, \tag{S31}$$

where $\gamma \leq 1/L$, and $\langle \cdot, \cdot \rangle$ denotes the inner product. We now derive the quadratic upper bounds for the three terms $F(\boldsymbol{x})$, $R_c(\boldsymbol{x})$, and $R_a(\boldsymbol{x})$ separately.

Data-fidelity function $F(\boldsymbol{x})$. It has been shown in our previous paper that the data-fidelity function $F(\boldsymbol{x})$ is upper-bounded by a quadratic function with $L_F = \rho(\boldsymbol{A}^\mathsf{H}\boldsymbol{A})$ [17].

Complex amplitude regularization function $R_c(\boldsymbol{x})$. The Wirtinger gradient is given by

$$\nabla_{\boldsymbol{x}} R_c(\boldsymbol{x}) = \lambda_c \boldsymbol{D}^\mathsf{T}\boldsymbol{D}\boldsymbol{x}. \tag{S32}$$

For any $\boldsymbol{x}_1, \boldsymbol{x}_2 \in \mathbb{C}^n$, we have

$$\begin{aligned} \|\nabla_{\boldsymbol{x}} R_c(\boldsymbol{x}_1) - \nabla_{\boldsymbol{x}} R_c(\boldsymbol{x}_2)\|_2 &= \lambda_c \|\boldsymbol{D}^\mathsf{T}\boldsymbol{D}(\boldsymbol{x}_1 - \boldsymbol{x}_2)\|_2 \\ &\leq \lambda_c \|\boldsymbol{D}^\mathsf{T}\boldsymbol{D}\|_2 \|\boldsymbol{x}_1 - \boldsymbol{x}_2\|_2 = \lambda_c \rho(\boldsymbol{D}^\mathsf{T}\boldsymbol{D}) \|\boldsymbol{x}_1 - \boldsymbol{x}_2\|_2, \end{aligned} \tag{S33}$$

which implies that $\nabla_{\boldsymbol{x}} R_c(\boldsymbol{x})$ has a Lipschitz upper bound of $L_{R_c} = \lambda_c \rho(\boldsymbol{D}^\mathsf{T}\boldsymbol{D})$.

Amplitude regularization function $R_a(\boldsymbol{x})$. $R_a(\boldsymbol{x})$ is nonsmooth for $\boldsymbol{x} \in Z$, where $Z \overset{\text{def}}{=} \{\boldsymbol{x} \in \mathbb{C}^n : \exists\, 1 \leq i \leq n, \text{s.t. } |x_i| = 0\}$. For $\boldsymbol{x} \in \mathbb{C}^n \backslash Z$, recall that the Wirtinger gradient is given by

$$\nabla_{\boldsymbol{x}} R_a(\boldsymbol{x}) = \lambda_a \text{diag}\left(\frac{\boldsymbol{x}}{|\boldsymbol{x}|}\right) \boldsymbol{D}^{\mathsf{T}} \boldsymbol{D} |\boldsymbol{x}|. \tag{S34}$$

The complex Hessian is defined as

$$\nabla^2 R_a(\boldsymbol{x}) = \boldsymbol{H}_{\tilde{\boldsymbol{x}}\tilde{\boldsymbol{x}}} = \begin{pmatrix} \boldsymbol{H}_{\boldsymbol{x}\boldsymbol{x}} & \boldsymbol{H}_{\bar{\boldsymbol{x}}\boldsymbol{x}} \\ \boldsymbol{H}_{\boldsymbol{x}\bar{\boldsymbol{x}}} & \boldsymbol{H}_{\bar{\boldsymbol{x}}\bar{\boldsymbol{x}}} \end{pmatrix}, \tag{S35}$$

where the four second-order partial derivatives are calculated as follows:

$$\begin{aligned}
\boldsymbol{H}_{\boldsymbol{x}\boldsymbol{x}} =& \frac{\partial}{\partial \boldsymbol{x}} \left(\frac{\partial R_a(\boldsymbol{x})}{\partial \boldsymbol{x}}\right)^{\mathsf{H}} \\
=& \lambda_a \frac{\partial}{\partial \boldsymbol{x}} \left(\text{diag}\left(\frac{\boldsymbol{x}}{|\boldsymbol{x}|}\right) \boldsymbol{D}^{\mathsf{T}} \boldsymbol{D} |\boldsymbol{x}|\right) \\
=& \frac{\lambda_a}{2} \left(\text{diag}\left(\frac{\boldsymbol{x}}{|\boldsymbol{x}|}\right) \boldsymbol{D}^{\mathsf{T}} \boldsymbol{D} \text{diag}\left(\frac{\bar{\boldsymbol{x}}}{|\boldsymbol{x}|}\right) + \text{diag}\left(\frac{\boldsymbol{D}^{\mathsf{T}} \boldsymbol{D} |\boldsymbol{x}|}{|\boldsymbol{x}|}\right)\right) \\
=& \frac{\lambda_a}{2} \text{diag}\left(\frac{\boldsymbol{x}}{|\boldsymbol{x}|}\right) \left(\boldsymbol{D}^{\mathsf{T}} \boldsymbol{D} + \text{diag}\left(\frac{\boldsymbol{D}^{\mathsf{T}} \boldsymbol{D} |\boldsymbol{x}|}{|\boldsymbol{x}|}\right)\right) \text{diag}\left(\frac{\bar{\boldsymbol{x}}}{|\boldsymbol{x}|}\right), \qquad \text{(S36)} \\
\boldsymbol{H}_{\bar{\boldsymbol{x}}\boldsymbol{x}} =& \frac{\partial}{\partial \bar{\boldsymbol{x}}} \left(\frac{\partial R_a(\boldsymbol{x})}{\partial \boldsymbol{x}}\right)^{\mathsf{H}} \\
=& \lambda_a \frac{\partial}{\partial \bar{\boldsymbol{x}}} \left(\text{diag}\left(\frac{\boldsymbol{x}}{|\boldsymbol{x}|}\right) \boldsymbol{D}^{\mathsf{T}} \boldsymbol{D} |\boldsymbol{x}|\right) \\
=& \frac{\lambda_a}{2} \left(\text{diag}\left(\frac{\boldsymbol{x}}{|\boldsymbol{x}|}\right) \boldsymbol{D}^{\mathsf{T}} \boldsymbol{D} \text{diag}\left(\frac{\boldsymbol{x}}{|\boldsymbol{x}|}\right) - \text{diag}\left(\frac{\boldsymbol{x}^2}{|\boldsymbol{x}|^3}\right) \text{diag}\left(\boldsymbol{D}^{\mathsf{T}} \boldsymbol{D} |\boldsymbol{x}|\right)\right) \\
=& \frac{\lambda_a}{2} \text{diag}\left(\frac{\boldsymbol{x}}{|\boldsymbol{x}|}\right) \left(\boldsymbol{D}^{\mathsf{T}} \boldsymbol{D} - \text{diag}\left(\frac{\boldsymbol{D}^{\mathsf{T}} \boldsymbol{D} |\boldsymbol{x}|}{|\boldsymbol{x}|}\right)\right) \text{diag}\left(\frac{\boldsymbol{x}}{|\boldsymbol{x}|}\right), \qquad \text{(S37)} \\
\boldsymbol{H}_{\boldsymbol{x}\bar{\boldsymbol{x}}} =& \frac{\partial}{\partial \boldsymbol{x}} \left(\frac{\partial R_a(\boldsymbol{x})}{\partial \bar{\boldsymbol{x}}}\right)^{\mathsf{H}} = \boldsymbol{H}_{\bar{\boldsymbol{x}}\boldsymbol{x}}^{\mathsf{H}} \\
=& \frac{\lambda_a}{2} \left(\text{diag}\left(\frac{\bar{\boldsymbol{x}}}{|\boldsymbol{x}|}\right) \boldsymbol{D}^{\mathsf{T}} \boldsymbol{D} \text{diag}\left(\frac{\bar{\boldsymbol{x}}}{|\boldsymbol{x}|}\right) - \text{diag}\left(\frac{\bar{\boldsymbol{x}}^2}{|\boldsymbol{x}|^3}\right) \text{diag}\left(\boldsymbol{D}^{\mathsf{T}} \boldsymbol{D} |\boldsymbol{x}|\right)\right) \\
=& \frac{\lambda_a}{2} \text{diag}\left(\frac{\bar{\boldsymbol{x}}}{|\boldsymbol{x}|}\right) \left(\boldsymbol{D}^{\mathsf{T}} \boldsymbol{D} - \text{diag}\left(\frac{\boldsymbol{D}^{\mathsf{T}} \boldsymbol{D} |\boldsymbol{x}|}{|\boldsymbol{x}|}\right)\right) \text{diag}\left(\frac{\bar{\boldsymbol{x}}}{|\boldsymbol{x}|}\right), \qquad \text{(S38)} \\
\boldsymbol{H}_{\bar{\boldsymbol{x}}\bar{\boldsymbol{x}}} =& \frac{\partial}{\partial \bar{\boldsymbol{x}}} \left(\frac{\partial R_a(\boldsymbol{x})}{\partial \bar{\boldsymbol{x}}}\right)^{\mathsf{H}} = \boldsymbol{H}_{\boldsymbol{x}\boldsymbol{x}}^{\mathsf{T}} \\
=& \frac{\lambda_a}{2} \left(\text{diag}\left(\frac{\bar{\boldsymbol{x}}}{|\boldsymbol{x}|}\right) \boldsymbol{D}^{\mathsf{T}} \boldsymbol{D} \text{diag}\left(\frac{\boldsymbol{x}}{|\boldsymbol{x}|}\right) + \text{diag}\left(\frac{\boldsymbol{D}^{\mathsf{T}} \boldsymbol{D} |\boldsymbol{x}|}{|\boldsymbol{x}|}\right)\right)
\end{aligned}$$

$$=\frac{\lambda_a}{2}\mathrm{diag}\left(\frac{\bar{\boldsymbol{x}}}{|\boldsymbol{x}|}\right)\left(\boldsymbol{D}^{\mathsf{T}}\boldsymbol{D}+\mathrm{diag}\left(\frac{\boldsymbol{D}^{\mathsf{T}}\boldsymbol{D}|\boldsymbol{x}|}{|\boldsymbol{x}|}\right)\right)\mathrm{diag}\left(\frac{\boldsymbol{x}}{|\boldsymbol{x}|}\right). \tag{S39}$$

Lemma 5. *For any $\boldsymbol{x}\in\mathbb{C}^n\backslash Z$, the Lipschitz constant for the gradient of the amplitude regularization function $\nabla_{\boldsymbol{x}}R_a(\boldsymbol{x})$ is bounded above by $L_{Ra}=\lambda_a\rho(\boldsymbol{D}^{\mathsf{T}}\boldsymbol{D})$.*

Proof. We only need to prove that for any $\tau>\lambda_a\rho(\boldsymbol{D}^{\mathsf{T}}\boldsymbol{D})$, we have

$$\boldsymbol{G}\equiv\tau\boldsymbol{I}-\boldsymbol{H}_{\tilde{\boldsymbol{x}}\tilde{\boldsymbol{x}}}=\begin{pmatrix}\tau\boldsymbol{I}-\boldsymbol{H}_{\boldsymbol{x}\boldsymbol{x}} & -\boldsymbol{H}_{\bar{\boldsymbol{x}}\boldsymbol{x}}\\ -\boldsymbol{H}_{\boldsymbol{x}\bar{\boldsymbol{x}}} & \tau\boldsymbol{I}-\boldsymbol{H}_{\bar{\boldsymbol{x}}\bar{\boldsymbol{x}}}\end{pmatrix}\succ\boldsymbol{0}. \tag{S40}$$

Denoting $\boldsymbol{G}_{11}$, $\boldsymbol{G}_{12}$, $\boldsymbol{G}_{21}$, $\boldsymbol{G}_{22}\in\mathbb{C}^{n\times n}$ as the four block matrices of $\boldsymbol{G}$, we have

$$\begin{aligned}\boldsymbol{G}_{11}=&\tau\boldsymbol{I}-\frac{\lambda_a}{2}\mathrm{diag}\left(\frac{\boldsymbol{x}}{|\boldsymbol{x}|}\right)\left(\boldsymbol{D}^{\mathsf{T}}\boldsymbol{D}+\mathrm{diag}\left(\frac{\boldsymbol{D}^{\mathsf{T}}\boldsymbol{D}|\boldsymbol{x}|}{|\boldsymbol{x}|}\right)\right)\mathrm{diag}\left(\frac{\bar{\boldsymbol{x}}}{|\boldsymbol{x}|}\right)\\ \succ&\frac{\lambda_a}{2}\mathrm{diag}\left(\frac{\boldsymbol{x}}{|\boldsymbol{x}|}\right)\left(2\rho(\boldsymbol{D}^{\mathsf{T}}\boldsymbol{D})\boldsymbol{I}-\boldsymbol{D}^{\mathsf{T}}\boldsymbol{D}-\mathrm{diag}\left(\frac{\boldsymbol{D}^{\mathsf{T}}\boldsymbol{D}|\boldsymbol{x}|}{|\boldsymbol{x}|}\right)\right)\mathrm{diag}\left(\frac{\bar{\boldsymbol{x}}}{|\boldsymbol{x}|}\right)\\ \succeq&\frac{\lambda_a}{2}\mathrm{diag}\left(\frac{\boldsymbol{x}}{|\boldsymbol{x}|}\right)\left(\rho(\boldsymbol{D}^{\mathsf{T}}\boldsymbol{D})\boldsymbol{I}-\mathrm{diag}\left(\frac{\boldsymbol{D}^{\mathsf{T}}\boldsymbol{D}|\boldsymbol{x}|}{|\boldsymbol{x}|}\right)\right)\mathrm{diag}\left(\frac{\bar{\boldsymbol{x}}}{|\boldsymbol{x}|}\right)\\ \overset{(a)}{\succeq}&\boldsymbol{0},\end{aligned} \tag{S41}$$

where (a) holds according to Lemma 4. And according to Lemma 1(i) and Lemma 1(ii), we have

$$\begin{aligned}&\boldsymbol{G}_{21}\boldsymbol{G}_{11}^{-1}\boldsymbol{G}_{12}\prec\boldsymbol{G}_{21}\frac{2}{\lambda_a}\mathrm{diag}\left(\frac{\boldsymbol{x}}{|\boldsymbol{x}|}\right)\left(\rho(\boldsymbol{D}^{\mathsf{T}}\boldsymbol{D})\boldsymbol{I}-\mathrm{diag}\left(\frac{\boldsymbol{D}^{\mathsf{T}}\boldsymbol{D}|\boldsymbol{x}|}{|\boldsymbol{x}|}\right)\right)^{-1}\mathrm{diag}\left(\frac{\bar{\boldsymbol{x}}}{|\boldsymbol{x}|}\right)\boldsymbol{G}_{12}\\ &=\frac{\lambda_a}{2}\mathrm{diag}\left(\frac{\bar{\boldsymbol{x}}}{|\boldsymbol{x}|}\right)\left(\boldsymbol{D}^{\mathsf{T}}\boldsymbol{D}-\mathrm{diag}\left(\frac{\boldsymbol{D}^{\mathsf{T}}\boldsymbol{D}|\boldsymbol{x}|}{|\boldsymbol{x}|}\right)\right)\left(\rho(\boldsymbol{D}^{\mathsf{T}}\boldsymbol{D})\boldsymbol{I}-\mathrm{diag}\left(\frac{\boldsymbol{D}^{\mathsf{T}}\boldsymbol{D}|\boldsymbol{x}|}{|\boldsymbol{x}|}\right)\right)^{-1}\\ &\quad\times\left(\boldsymbol{D}^{\mathsf{T}}\boldsymbol{D}-\mathrm{diag}\left(\frac{\boldsymbol{D}^{\mathsf{T}}\boldsymbol{D}|\boldsymbol{x}|}{|\boldsymbol{x}|}\right)\right)\mathrm{diag}\left(\frac{\boldsymbol{x}}{|\boldsymbol{x}|}\right)\\ &\overset{(b)}{\preceq}\frac{\lambda_a}{2}\mathrm{diag}\left(\frac{\bar{\boldsymbol{x}}}{|\boldsymbol{x}|}\right)\left(\rho(\boldsymbol{D}^{\mathsf{T}}\boldsymbol{D})\boldsymbol{I}-\mathrm{diag}\left(\frac{\boldsymbol{D}^{\mathsf{T}}\boldsymbol{D}|\boldsymbol{x}|}{|\boldsymbol{x}|}\right)\right)\left(\rho(\boldsymbol{D}^{\mathsf{T}}\boldsymbol{D})\boldsymbol{I}-\mathrm{diag}\left(\frac{\boldsymbol{D}^{\mathsf{T}}\boldsymbol{D}|\boldsymbol{x}|}{|\boldsymbol{x}|}\right)\right)^{-1}\\ &\quad\times\left(\rho(\boldsymbol{D}^{\mathsf{T}}\boldsymbol{D})\boldsymbol{I}-\mathrm{diag}\left(\frac{\boldsymbol{D}^{\mathsf{T}}\boldsymbol{D}|\boldsymbol{x}|}{|\boldsymbol{x}|}\right)\right)\mathrm{diag}\left(\frac{\boldsymbol{x}}{|\boldsymbol{x}|}\right)\\ &=\frac{\lambda_a}{2}\mathrm{diag}\left(\frac{\bar{\boldsymbol{x}}}{|\boldsymbol{x}|}\right)\left(\rho(\boldsymbol{D}^{\mathsf{T}}\boldsymbol{D})\boldsymbol{I}-\mathrm{diag}\left(\frac{\boldsymbol{D}^{\mathsf{T}}\boldsymbol{D}|\boldsymbol{x}|}{|\boldsymbol{x}|}\right)\right)\mathrm{diag}\left(\frac{\boldsymbol{x}}{|\boldsymbol{x}|}\right)\\ &\preceq\frac{\lambda_a}{2}\mathrm{diag}\left(\frac{\bar{\boldsymbol{x}}}{|\boldsymbol{x}|}\right)\left(2\rho(\boldsymbol{D}^{\mathsf{T}}\boldsymbol{D})\boldsymbol{I}-\boldsymbol{D}^{\mathsf{T}}\boldsymbol{D}-\mathrm{diag}\left(\frac{\boldsymbol{D}^{\mathsf{T}}\boldsymbol{D}|\boldsymbol{x}|}{|\boldsymbol{x}|}\right)\right)\mathrm{diag}\left(\frac{\boldsymbol{x}}{|\boldsymbol{x}|}\right)\\ &\prec\tau\boldsymbol{I}-\frac{\lambda_a}{2}\mathrm{diag}\left(\frac{\bar{\boldsymbol{x}}}{|\boldsymbol{x}|}\right)\left(\boldsymbol{D}^{\mathsf{T}}\boldsymbol{D}+\mathrm{diag}\left(\frac{\boldsymbol{D}^{\mathsf{T}}\boldsymbol{D}|\boldsymbol{x}|}{|\boldsymbol{x}|}\right)\right)\mathrm{diag}\left(\frac{\boldsymbol{x}}{|\boldsymbol{x}|}\right)\end{aligned}$$

$$= \boldsymbol{G}_{22}, \tag{S42}$$

where (b) holds because of Lemma 2. Therefore, according to Lemma 3, $\boldsymbol{G}$ is positive-definite. This implies that for $\boldsymbol{x} \in \mathbb{C}^n \backslash Z$ the Lipschitz constant of ∇R_a is upper-bounded: $L_{Ra} \leq \lambda_a \rho(\boldsymbol{D}^\mathsf{T}\boldsymbol{D})$. $\square$

The above Lemma implies that $R_a(\boldsymbol{x})$ is upper-bounded by a quadratic function for all $\boldsymbol{x} \in \mathbb{C}^n \backslash Z$. The following lemma states that $R_a(\boldsymbol{x})$ is in fact globally upper-bounded by the same quadratic function for all $\boldsymbol{x} \in \mathbb{C}^n$.

Lemma 6. *Given any $\boldsymbol{z} \in \mathbb{C}^n$, the amplitude regularization term $R_a(\boldsymbol{x})$ is upper-bounded by a quadratic function $Q_{R_a}(\boldsymbol{x})$:*

$$R_a(\boldsymbol{x}) \leq Q_{R_a}(\boldsymbol{x}) \stackrel{\text{def}}{=} R_a(\boldsymbol{z}) + \langle \nabla R_a(\boldsymbol{z}), \hat{\boldsymbol{x}} - \hat{\boldsymbol{z}} \rangle + \frac{L_{R_a}}{2} \|\hat{\boldsymbol{x}} - \hat{\boldsymbol{z}}\|_2^2. \tag{S43}$$

Proof. Let $\Delta\boldsymbol{x} = \boldsymbol{x} - \boldsymbol{z}$, then either of the two following cases occurs:

1) The line between $\boldsymbol{x}$ and $\boldsymbol{z}$ does not pass through any nonsmooth points, i.e., $\boldsymbol{z} + \alpha\Delta\boldsymbol{x} \in \mathbb{C}\backslash Z, \forall \alpha \in [0,1]$, or $\boldsymbol{x}$ and $\boldsymbol{z}$ lie in the subspace, i.e., $\boldsymbol{z} + \alpha\Delta\boldsymbol{x} \in Z, \forall\alpha \in [0,1]$, the result is obtained directly according to the multivariate Taylor expansion of R_a:

$$\begin{aligned} R_a(\boldsymbol{x}) =& R_a(\boldsymbol{z}) + \langle \nabla R_a(\boldsymbol{z}), \hat{\boldsymbol{x}} - \hat{\boldsymbol{z}} \rangle + \frac{1}{2}(\hat{\boldsymbol{x}} - \hat{\boldsymbol{z}})^\mathsf{H} \nabla^2 R_a(\boldsymbol{u})(\hat{\boldsymbol{x}} - \hat{\boldsymbol{z}}) \\ \leq& R_a(\boldsymbol{z}) + \langle \nabla R_a(\boldsymbol{z}), \hat{\boldsymbol{x}} - \hat{\boldsymbol{z}} \rangle + \frac{L_{R_a}}{2} \|\hat{\boldsymbol{x}} - \hat{\boldsymbol{z}}\|_2^2 \\ =& Q_{R_a}(\boldsymbol{x}), \end{aligned} \tag{S44}$$

where $\boldsymbol{u}$ is a convex combination of $\boldsymbol{x}$ and $\boldsymbol{z}$.

2) The line between $\boldsymbol{x}$ and $\boldsymbol{z}$ passes through a finite number of nonsmooth points. For simplicity, we consider the case of passing through a single nonsmooth point indexed by i, that is, we have

$$|x_i| = |z_i + \alpha^\star \Delta x_i| = 0, \tag{S45}$$

for some $0 < \alpha^\star < 1$. According to 1), for any $0 \leq \alpha \leq \alpha^\star$, $R_a(\boldsymbol{x})$ is upper-bounded by $Q_{R_a}(\boldsymbol{x})$. We now prove that this also holds for any $\alpha^\star < \alpha < 1$. $R_a(\boldsymbol{x})$ can be written as a function over α for any point that lies on the line between $\boldsymbol{x}$ ad $\boldsymbol{z}$:

$$\begin{aligned} g(\alpha) =& R_a(\boldsymbol{z} + \alpha\Delta\boldsymbol{x}) = \lambda_a(|x_i| - |x_j|)^2 + \lambda_a \sum_{k,l}(|x_k| - |x_l|)^2 \\ =& \lambda_a(|z_i + \alpha\Delta x_i| - |x_j|)^2 + \lambda_a \sum_{k,l}(|x_k| - |x_l|)^2 \\ =& \lambda_a((\alpha - \alpha^\star)|\Delta x_i| - |x_j|)^2 + \lambda_a \sum_{k,l}(|x_k| - |x_l|)^2 \end{aligned}$$

$$\leq \lambda_a((\alpha - \alpha^\star)|\Delta x_i| + |x_j|)^2 + \lambda_a \sum_{k,l} (|x_k| - |x_l|)^2$$

$$\leq h(\alpha), \tag{S46}$$

where j denotes the pixel index adjacent to i, and $h(\alpha) \overset{\text{def}}{=} Q_{R_a}(\boldsymbol{z} + \alpha \Delta \boldsymbol{x})$. As a result, we have

$$R_a(\boldsymbol{x}) = R_a(\boldsymbol{z} + \Delta \boldsymbol{x}) = g(1) \leq h(1) = Q_{R_a}(\boldsymbol{x}). \tag{S47}$$

The above derivation can be easily extended to the case of multiple nonsmooth points.

With this, we conclude that for any $\boldsymbol{x} \in \mathbb{C}^n$, we have

$$R_a(\boldsymbol{x}) \leq Q_{R_a}(\boldsymbol{x}), \tag{S48}$$

which completes the proof. □

Based on the above analysis, we can establish the convergence theorem of the algorithm as summarized below. The proof directly follows from the results in Ref. [18]. Note that owing to the nonconvexity of the phase retrieval problem, only proof for the basic proximal gradient algorithm is presented, yet we empirically observed stable convergence for the accelerated algorithm as well.

Theorem 1. *The basic proximal gradient algorithm (with $\beta_i = 0$ in Eq. (9)) converges to a stationary point using a fixed step size that satisfies*

$$\gamma \leq \frac{1}{\rho(\boldsymbol{A}^\mathsf{H}\boldsymbol{A}) + \lambda_a \rho(\boldsymbol{D}^\mathsf{T}\boldsymbol{D}) + \lambda_c \rho(\boldsymbol{D}^\mathsf{T}\boldsymbol{D})}. \tag{S49}$$

7 Ptychographic reconstruction algorithms

7.1 Blind ptychographic reconstruction for DOE calibration

Recall from the main text that the blind ptychographic reconstruction problem for the joint recovery of DOE transmission function and the illumination wavefront is given by

$$\hat{\boldsymbol{m}}, \hat{\boldsymbol{p}} = \underset{\boldsymbol{m},\boldsymbol{p}}{\operatorname{argmin}} \left\{ \frac{1}{K} \sum_{k=1}^{K} J_k(\boldsymbol{m}, \boldsymbol{p}) = \frac{1}{K} \sum_{k=1}^{K} \| |\boldsymbol{Q} \operatorname{diag}(\boldsymbol{m}) \boldsymbol{T}_k \boldsymbol{p}| - \boldsymbol{y}_k \|_2^2 \right\}. \tag{S50}$$

Following the notation convention of ptychographic phase retrieval, for the k-th measurement, we define the shifted probe as $\boldsymbol{p}_k = \boldsymbol{T}_k \boldsymbol{p}$ and the exit wave as $\boldsymbol{e}_k = \operatorname{diag}(\boldsymbol{m}) \boldsymbol{p}_k$. The Wirtinger gradients are

$$\nabla_{\boldsymbol{m}} J_k = \operatorname{diag}(\bar{\boldsymbol{p}}_k) \boldsymbol{Q}^\mathsf{H} \operatorname{diag}\left(\frac{\boldsymbol{Q}\boldsymbol{e}_k}{|\boldsymbol{Q}\boldsymbol{e}_k|} \right) (|\boldsymbol{Q}\boldsymbol{e}_k| - \boldsymbol{y}_k), \tag{S51}$$

$$\nabla_{\boldsymbol{p}_k} J_k = \operatorname{diag}(\bar{\boldsymbol{m}}) \boldsymbol{Q}^\mathsf{H} \operatorname{diag}\left(\frac{\boldsymbol{Q}\boldsymbol{e}_k}{|\boldsymbol{Q}\boldsymbol{e}_k|} \right) (|\boldsymbol{Q}\boldsymbol{e}_k| - \boldsymbol{y}_k), \tag{S52}$$

$$\nabla_{\boldsymbol{p}} J_k = \boldsymbol{T}_k^{\mathsf{H}} \nabla_{\boldsymbol{p}_k} J_k. \tag{S53}$$

Inspired by the rPIE algorithm, we employ a modified stochastic gradient descent algorithm with spatially varying weights [19]:

$$\boldsymbol{m} \leftarrow \boldsymbol{m} - \mathrm{diag}(\boldsymbol{w}_{\alpha_m}(\boldsymbol{p})) \nabla_{\boldsymbol{m}} J_k, \tag{S54}$$

$$\boldsymbol{p} \leftarrow \boldsymbol{p} - (\nabla_{\boldsymbol{p}} \boldsymbol{p}_k) \mathrm{diag}(\boldsymbol{w}_{\alpha_p}(\boldsymbol{m})) \nabla_{\boldsymbol{p}} J_k, \tag{S55}$$

where the weight function is defined as

$$\boldsymbol{w}_{\alpha}(\boldsymbol{u}) = \frac{\mathbf{1}}{(1-\alpha)|\boldsymbol{u}|^2 + \alpha \max(|\boldsymbol{u}|^2)}, \tag{S56}$$

where α is a tunable parameter. In this work, we set $\alpha_m = 0.8$ and $\alpha_p = 1$. During the reconstruction, $\boldsymbol{m}$ and $\boldsymbol{p}$ are sequentially updated with respective to each measurement with k following a random order. We discovered that initializing the DOE profile as the designed value could boost the convergence and reduce the number of iterations required.

7.2 Non-blind ptychographic wavefront reconstruction

Once the DOE transmission function has been calibrated, the relative position between the DOE and the image sensor remains fixed throughout the experiment. As a result, non-blind ptychographic reconstruction is performed for each wavefront:

$$\hat{\boldsymbol{p}} = \underset{\boldsymbol{p}}{\mathrm{argmin}} \left\{ \frac{1}{K} \sum_{k=1}^{K} J_k(\boldsymbol{p}) = \frac{1}{K} \sum_{k=1}^{K} \| |\boldsymbol{Q} \mathrm{diag}(\boldsymbol{m}) \boldsymbol{T}_k \boldsymbol{p}| - \boldsymbol{y}_k \|_2^2 \right\}. \tag{S57}$$

The objective function is essentially the same as Eq. (S50) except that $\boldsymbol{m}$ is treated as a known parameter. A stochastic gradient descent algorithm is employed:

$$\boldsymbol{p} \leftarrow \boldsymbol{p} - \gamma \nabla_{\boldsymbol{p}} J_k, \tag{S58}$$

which proceeds following a random order.

Supplementary Videos

Supplementary Video 1 Visualization of the autofocusing process. The propagation distance d increases from $d = 0$ mm to the focal position of $d = 14.5$ mm with a step size of 0.1 mm. The amplitude and phase of the wavefront are shown for each propagation distance.

References

[1] Parikh, N., Boyd, S., *et al.*: Proximal algorithms. Foundations and Trends® in Optimization **1**(3), 127–239 (2014) https://doi.org/10.1561/2400000003

[2] https://github.com/THUHoloLab/SAFARI

[3] Goodman, J.W.: Introduction to Fourier Optics. Roberts and Company Publishers, Greenwood Village, Colorado (2005)

[4] Agustsson, E., Timofte, R.: NTIRE 2017 challenge on single image super-resolution: Dataset and study. In: The IEEE Conference on Computer Vision and Pattern Recognition (CVPR) Workshops (2017). https://doi.org/10.1109/CVPRW.2017.150

[5] Schmidt, J.D.: Numerical Simulation of Optical Wave Propagation with Examples in MATLAB. SPIE, Bellingham, Washington (2010). https://doi.org/10.1117/3.866274

[6] Huang, Y., Zhang, H., Liu, T., Lin, A., Zhang, F.: Single-shot diffractive spectrometer for photonic orbital angular momentum. APL Photonics **10**(2), 026104 (2025) https://doi.org/10.1063/5.0242874

[7] Zou, C., Veetil, S.P., Rong, N., Jiang, Z., Liu, C., Zhu, J.: Reference-free mode decomposition of few-mode fibers via coherent modulation imaging. Optics Letters **50**(11), 3648–3651 (2025) https://doi.org/10.1364/OL.553224

[8] Berto, P., Rigneault, H., Guillon, M.: Wavefront sensing with a thin diffuser. Optics Letters **42**(24), 5117–5120 (2017) https://doi.org/10.1364/OL.42.005117

[9] Wu, T., Berto, P., Guillon, M.: Reference-less complex wavefields characterization with a high-resolution wavefront sensor. Applied Physics Letters **118**(25), 251102 (2021) https://doi.org/10.1063/5.0050036

[10] Wu, T., Zhang, Y., Blochet, B., Arjmand, P., Berto, P., Guillon, M.: Single-shot digital optical fluorescence phase conjugation through forward multiple-scattering samples. Science Advances **10**(3), 1120 (2024) https://doi.org/10.1126/sciadv.adi1120

[11] Zhang, Q., Sui, Y., Rothe, S., Koukourakis, N., Czarske, J.: Reference-less decomposition of highly multimode fibers using a physics-driven neural network (2024) https://doi.org/10.21203/rs.3.rs-5110336/v1

[12] Oh, J., Lee, K., Park, Y.: Single-shot reference-free holographic imaging using a liquid crystal geometric phase diffuser. Laser & Photonics Reviews **16**(3), 2100559 (2022) https://doi.org/10.1002/lpor.202100559

[13] Baffou, G.: Quantitative phase microscopy using quadriwave lateral shearing interferometry (QLSI): principle, terminology, algorithm and grating shadow description. Journal of Physics D: Applied Physics **54**(29), 294002 (2021) https://doi.org/10.1088/1361-6463/abfbf9

[14] Farré, A., Shayegan, M., López-Quesada, C., Blab, G.A., Montes-Usategui, M., Forde, N.R., Martín-Badosa, E.: Positional stability of holographic optical traps. Optics Express **19**(22), 21370–21384 (2011) https://doi.org/10.1364/OE.19.021370

[15] Kreutz-Delgado, K.: The complex gradient operator and the CR-calculus. arXiv preprint arXiv:0906.4835 (2009) https://doi.org/10.48550/arXiv.0906.4835

[16] Horn, R.A., Johnson, C.R.: Matrix Analysis. Cambridge University Press, New York, New York (2012). https://doi.org/10.1017/CBO9780511810817

[17] Gao, Y., Cao, L.: Iterative projection meets sparsity regularization: towards practical single-shot quantitative phase imaging with in-line holography. Light: Advanced Manufacturing **4**(1), 37–53 (2023) https://doi.org/10.37188/lam.2023.006

[18] Beck, A.: First-Order Methods in Optimization. SIAM, Philadelphia, Pennsylvania (2017). https://doi.org/10.1137/1.9781611974997

[19] Maiden, A., Johnson, D., Li, P.: Further improvements to the ptychographical iterative engine. Optica **4**(7), 736–745 (2017) https://doi.org/10.1364/OPTICA.4.000736
